# Approaches for GC-HRMS Screening of Organic Microcontaminants: GC-APCI-IMS-QTOF versus GC-EI-QOrbitrap

**DOI:** 10.1021/acs.est.4c11032

**Published:** 2025-01-31

**Authors:** David Izquierdo-Sandoval, Juan Vicente Sancho, Félix Hernández, Tania Portoles

**Affiliations:** Environmental and Public Health Analytical Chemistry, Research Institute for Pesticides and Water (IUPA), 16748 Universitat Jaume I, Av. Sos Baynat S/N, Castellón de la Plana 12071, Spain

**Keywords:** target, suspect and non-target screening, ion mobility, GC-HRMS, environment and food safety, identification

## Abstract

This study explores the capabilities of GC-APCI-IMS-QTOF MS and GC-EI-QOrbitrap MS in screening applications and different strategies for wide-scope screening of organic microcontaminants using target suspect and nontarget approaches. On one side, GC-APCI-IMS-QTOF MS excels at preserving molecular information and adds ion mobility separation, facilitating screening through the list of componentized features containing accurate mass, retention time, CCS, and fragmentation data. On the other side, the extensive and robust fragmentation of GC-EI-QOrbitrap MS allows the application of different strategies for target and nontarget approaches using the NIST library spectra. Our findings revealed that GC-EI-QOrbitrap MS is more sensitive in target approaches. Automated workflows for suspect screening in GC-APCI-IMS-QTOF MS minimize false annotations but face challenges with false negatives due to in-source fragmentation and limitations when using *in silico* fragmentation tools. Conversely, a nontarget approach in GC-EI-QOrbitrap MS can reliably identify unknowns but results in more false annotations in complex matrices. This work highlights the strengths and limitations of each system and guides for their optimal application for wide-scope screening in environmental and food safety applications.

## Graphical abstract

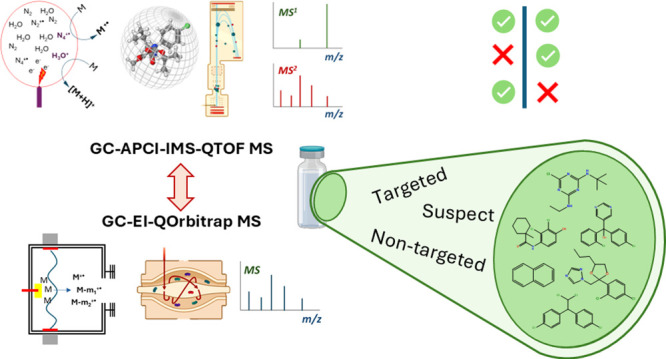

## 1 Introduction

Gas chromatography coupled to high-resolution mass spectrometry (GC-HRMS) is a robust analytical setup widely trusted for its consistency in monitoring nonpolar, volatile, and thermally stable organic micropollutants (OMPs) in complex matrixes.^1^ Time of flight (TOF) and Orbitrap mass analyzers, with their ability to acquire full-spectrum accurate mass data, can virtually screen any ionizable compound in a single analysis with reasonable sensitivity.^2^ The increased mass accuracy and resolving power significantly reduce interferences, prioritizing a candidate’s selection at a given mass. Moreover, this comprehensive acquisition enables retrospective analysis of unexpected compounds in previously acquired data.^3^ These unique characteristics make hyphenated techniques (GC/LC-HRMS) not only versatile but also intriguing methodologies for developing screening strategies and identifying and confirming OMPs in a wide range of environmental and food safety contexts.^4,5^

Electron ionization (EI) is the most widely implemented ionization source in GC-MS due to its robustness and reproducibility in ionization and fragmentation.^6^ The properties of EI-derived spectra have led to the development of universal spectral libraries, such as the National Institute of Standards and Technology (NIST), which contains unique EI-MS spectra and retention indexes (RI) for hundreds of thousands of compounds.^7^ GC-EI-TOF-MS has proven to be a potent tool for screening strategies based on both nominal spectral libraries^8^ and exact mass spectral libraries.^9^ In recent years, GC-EI-Orbitrap MS has become increasingly popular as a screening tool due to its superior mass accuracy (≤1 mDa), resolving power (around 120.000 fwhm at 272 *m*/*z*), acquisition rate, and sensitivity.^10–13^ By using hybrid mass analyzers (e.g., QTOF and QOrbitrap), it is possible to combine the full-scan MS acquisition with MS/MS fragmentation. However, the extensive in-source fragmentation characteristic of EI produces excessive fragmentation in combination with tandem MS acquisition modes, making structural characterization more challenging. Under these circumstances, the possibility of using universal EI-MS spectral libraries facilitates the identification in full-scan mode.^14^

In contrast to EI hard ionization, the soft ionization promoted by atmospheric pressure chemical ionization (APCI) preserves the molecular and quasi-molecular ion information, enabling different acquisition approaches in hybrid HRMS analyzers.^6^ The use of GC-APCI-QTOF MS under data-independent acquisition (DIA) mode allows obtaining structural information through the fragmentation of the ionized molecules by aligning low and high collision energy spectra at a specific retention time (e.g., MS^E^), producing fragmentation of the ions and resulting in valuable structure information for elucidation, identification, and confirmation of the compounds in a variety of sample matrixes.^15,16^ Furthermore, under specific APCI conditions, ion–molecule reactions can lead to complex rearrangements, offering additional structural information. These capabilities have been demonstrated in key classes of OMPs, including halogenated dibenzo-p-dioxins^17^ and organophosphate esters.^18^

Recently, ion mobility separation (IMS) has been implemented in GC-APCI-QTOF MS systems.^19,20^ IMS-HRMS provides several advantages to the screening approaches. First, ions are subjected to an additional separation, the drift time, based on their charge, shape, and size. Thus, it is possible to acquire drift-aligned MS^E^ spectra (HDMS^E^), which remove interferent ions in low and high-energy spectra.^19^ Second, the drift-derived collisional cross-section (CCS) parameter is reproducible between instruments with the same IMS technology and independent of the complexity of the matrix, the chromatographic conditions, and the ionization source.^21^ As a result, CCS can be included as an additional value in the criteria used to confirm candidate structures in nontarget workflows.^22,23^

When designing a screening approach, it is essential to consider the research question and the amount of information available about the compounds of interest.^24^ A target screening involves chromatographic, and MS (or MS/MS) information obtained from reference standards measured in-house. This has been applied using GC-APCI-QTOF MS,^25^ GC-APCI-IMS-QTOF MS,^19^ and GC-QOrbitrap MS.^26^ In recent years, software advancements for data treatment have sparked interest in more comprehensive approaches, not limited to monitoring a small set of target chemicals. Suspect screening (SS) and nontarget screening (NTS) have gained popularity for their ability to thoroughly explore a wide range of compounds. Although the borders between both terms are blurry, SS typically involves lists of exact masses and isotope patterns of different adducts based on the molecular formula of the compounds, whereas no previous structural information is available in NTS.^3^ Hyphenated chromatography-HRMS generates massive amounts of data, making the identification and confirmation process laborious in SS and NTS approaches. Therefore, a prioritization strategy is necessary to reduce the number of candidates and select only those compounds of relevance for the study.^27^

The objective of this work is to evaluate the potential of modern GC-APCI-IMS-QTOF MS and GC-EI-QOrbitrap MS configurations for monitoring GC-amenable OMPs in complex matrices in food safety and environmental fields. This study compares the two systems through a targeted approach, screening a predefined list of compounds spiked into samples. Subsequently, the capabilities of GC-APCI-IMS-QTOF MS for suspect screening and GC-EI-QOrbitrap MS for nontarget screening are explored using the same spiked samples. This dual approach highlights illustrative examples of the strengths and limitations of each configuration for screening applications in complex matrices.

## 2 Materials and Method

### 2.1 Reagents and Chemicals

Reference standards from 201 contaminants were purchased from different vendors, of which 162 were pesticides, 17 PAHs, 9 OPEs, five musks, one insect repellent, and 7 PCBs. Reference standards of pesticides, PCBs and PAHs were purchased from Dr. Ehrenstorfer (Augsburg, Germany). OPEs standard mixtures were purchased from Chiron (Trondheim, Norway). Musk standards were purchased from LGC Standards (Barcelona, Spain), and the insect repellent was from Sigma-Aldrich (Madrid, Spain). The complete list of contaminants can be found in Table S2. For pesticides, OPEs, musks, and insect repellent, standard solutions of 500 mg/L were prepared in acetone and stored in a freezer at −20 °C. PAHs were purchased at a concentration of 10 mg/L, and Mix PCB-3 was purchased at 100 mg/L; both were also stored in a freezer at −20 °C. QuEChERS commercial cleanup kits were purchased from Teknokroma (Barcelona, Spain). Each kit contains 50 mg of primary-secondary amine (PSA), 150 mg of anhydrous magnesium sulfate, and 50 mg of C18 in 2 mL microcentrifuge tubes for d-SPE. UHPLC-grade water was obtained from a Milli-Q water purification system (Ultramatic Plus GR, Wasserlab, Navarra, Spain). Acetone (pesticide residue analysis quality) and *n*-hexane (ultratrace quality) were purchased from Scharlab (Barcelona, Spain).

### 2.2 Selected Samples

To evaluate the screening performance of both GC-HRMS instruments in target, suspect, and nontarget approaches, nine different matrixes were spiked with a mix of 201 GC-amenable pollutants. The selected samples included six feed raw ingredients and three complete fish feed formulations. Fish feed 1 and ingredient 2 are based on alternative ingredients such as insect meal or algae oil, fish feed 2 and ingredients 1 and 6 are based on fish meals, and finally, fish feed 3 and ingredients 3, 4, and 5 are based on processed animal and plant proteins. Feed ingredients and aquafeeds were directly purchased from manufacturers.

### 2.3 Sample Treatment

Before extraction, samples were spiked at 50 μg kg^–1^ with a mix of 201 OMPs in hexane and then allowed to stand for 1 h to facilitate the interaction between the contaminants and the matrix. Previously, feed samples were homogenized with dry ice using a crushing machine. Briefly, 5 g of sample was accurately weighed in 50 mL centrifuge tubes and vortexed with 10 mL of acetonitrile. Next, 1 g of MgSO_4_ was added, and the mixture was shaken again for 30 s before centrifugation in 1893 rcf for 12 min. The supernatant was transferred to 15 mL centrifuge tubes and stored overnight in the freezer to promote protein precipitation and lipid deposition on the tube walls. Following this, 1 mL was transferred to Eppendorf tubes prepared for QuEChERS cleanup, containing 50 mg PSA, 150 mg MgSO_4_, and 50 mg C18. The mixture was vortexed for 30 s and then centrifuged at 12,557 rcf for 5 min. Subsequently, 600 μL of the supernatant was transferred to a glass tube and evaporated to dryness at 40 °C under a gentle nitrogen stream. Samples were reconstituted with 200 μL of hexane and stored in the freezer until injection into GC-HRMS.

### 2.4 Instrumentation

#### 2.4.1 GC-APCI-IMS-QTOF MS

A VION mass spectrometer (Waters Corporation, Manchester, UK) coupled to an Agilent 7890 N gas chromatograph (Palo Alto, CA, USA) through an APGC v2.0 interface was employed in positive APCI mode. GC separation and MS acquisition were achieved as described previously.^16^ Briefly, a fused silica DB 5MS capillary column of 30 m × 0.25 mm i.d. and a film thickness of 0.25 μm (J&W Scientific, Folson, CA, USA) was used. The temperature program started at 90 °C for 1 min, then increased to 315 for 45 min, and held for 4 min with a total runtime of 50 min. Helium 99.999% (Praxair, Spain) was used as carrier gas at a 4 mL/min flow. The injector was set at 280 °C in pulsed splitless mode (30 psi, 1 min) with injections of 1 μL. For the APCI, the corona discharge pin was operated at 2.0 μA, the cone voltage was set to 40 V, and the source temperature was set to 150 °C. N_2_ was used as an auxiliary (400 L/h), cone (150 L/h), and makeup gas (100 mL/min). Interface temperature was set to 315 °C. APCI was operated in two different configurations. Wet conditions promote protonation by facilitating the formation of water steam in the source. Dry conditions promote the formation of molecular ions by removing water traces before the analysis.

Before MS acquisition, ions were separated by their drift time using a traveling-wave ion mobility spectrometer (TWIMS) with a wave velocity of 250 m/s and a wave height ramp of 20–50 V. Then, a QTOF MS in high-definition (HD) MS^E^ mode, in the range of 50–1000 *m*/*z*, enables the acquisition of two independent acquisition functions with different collision energies: a collision energy of 6 eV for the low energy function (LE), and a ramp of 21–56 eV for the high energy function (HE). The scan times for both LE and HE functions were 0.25 s. Nitrogen (≥99.999%) was used as the drift and collision-induced dissociation (CID) gas. Internal mass calibration was performed using two GC-column bleeding ions as lock mass monitoring the molecular ions, *m*/*z* 355.06994/C_9_H_27_O_5_Si_5_^+^ and 223.06365/C_6_H_19_O_3_Si_3_^+^, respectively. The instrument was calibrated for *m*/*z* measurements and CCS calculation following the manufacturer’s instructions using a Z-Spray electrospray ionization source (Waters Corp.). MS data were acquired and processed using Waters’s UNIFI informatics platform (v 1.9).

#### 2.4.2 GC-EI-QOrbitrap MS

Data were acquired using a Thermo Scientific Q Exactive GC hybrid quadrupole-Orbitrap mass spectrometer. Sample injection was performed with a TriPlus RSH autosampler (Thermo Scientific, Bremen, Germany). The chromatographic separation was carried out with a Thermo Scientific TRACE 1310 GC with an HP-5MS column (Thermo Fisher Scientific, Palo Alto, CA, USA) of 30 m × 0.25 mm i.d. × 0.25 μm film thickness following the same temperature ramp as the one used in the GC-APCI-IMS-QTOF set up. Helium (99.999% purity) was used as carrier gas at a constant flow rate of 1 mL min^–1^. The injector temperature was set at 300 °C in split/splitless mode with a flow rate of 50 mL min^–1^, a purge time of 1.0 min, and an injection volume of 1 μL. C_8_–C_40_ alkane series was used for the external nonisothermal retention index (RI) in both GC-HRMS systems.

Data acquisition was performed in positive electron ionization (EI) at 70 eV, operating in full scan mode with a resolution of 60,000 fwhm at 272 *m*/*z*. Ion source and transfer line temperatures were set at 270 and 330 °C, respectively. The scanned range was 40 to 650 *m*/*z* at a scan rate of 3.7 scan/s. Nitrogen gas (Praxair, Spain) was used for the C-Trap supply, which ion handling used an automatic gain control (AGC) of 1 × 10,^6^ and the injection time (IT) was set to automatic. A calibration procedure was performed daily, using an internal calibration gas as a calibrant. Instrument control and data acquisition were achieved using Xcalibur 4.0 software (Thermo Scientific, Waltham, MA, USA).

### 2.5 Data Processing

#### 2.5.1 Target and Suspect Screening in GC-APCI-IMS-QTOF MS

Both target and suspect lists were imported into Waters UNIFI software (Waters Corporation) through spreadsheet and MOL files and incorporated into in-software screening libraries. Lock mass correction, background subtraction, peak picking, and deconvolution were performed automatically in the software, as summarized in Table S1. During sample analysis, compounds were tentatively annotated by comparing accurate mass measurements with the exact (theoretical) masses of the compounds in the libraries using a user-defined threshold of 5.0 mDa. Different filters were applied to this comprehensive assignment depending on the approach, as explained below. The steps varied from the most restrictive situations to those where some parameters could fail.

For target screening, a homemade database elaborated in our research group was used.^20^ It includes chromatography parameters (e.g., retention time), mobility (^TW^CCS), (de)­protonated and molecular exact mass, and HDMS^E^ fragmentation for 201 GC-amenable OMPs, for which standard solutions were previously injected into our equipment. The criteria for positive confirmation were based on the requirements described in European SANTE guidelines (SANTE/11312/2021) for HRMS analysis: at least two ions with mass accuracy ≤5 ppm (or ≤ ± 1 mDa if *m*/*z* < 200) and a S/*N* > 5, and a retention time tolerance of ±0.1 min.^28^ The confirmation of the identity was improved using ion mobility. A CCS tolerance of ±2% was established based on our previous study.^20^

To assess the performance of the suspect screening, a list of suspects was built for the same contaminants included in the database. This approach treated the compounds as suspects, considering only exact mass, Kovats retention indexes (RI), and utilizing in-silico computational tools. Retention times were calculated from Kovats’ RI on a nonpolar column (preferably DB-5MS) in the NIST23.^7^ NIST database containing experimental and estimated RIs for 193 and 8 contaminants, respectively. Since UNIFI software only works with retention time, it was necessary to convert RIs into equivalent retention time values for chromatographic conditions using a C_8_–C_40_ alkane series. Additionally, a Multiple Adaptative Regression Spline (MARS) model developed previously in our research group^29^ was used to predict the CCS of the (de)­protonated ions using as input the experimental CCS_H_ values collected in the homemade library elaborated in our research group.^20^ Table S2 contains the complete suspect screening list for GC-APCI-IMS-QTOF MS. Observed fragment peaks were matched to MOL-defined structures using the in-software ion structural tool MassFragment. Restrictive parameters included MS, RT and CCS criteria. For MS, an accuracy of 5 ppm (or ±1 mDa if *m*/*z* < 200) from the theoretical *m*/*z* of precursor ions ([M + H]^+^ and M^+^•) was applied, requiring at least one in-software structurally explained fragment (mass error <2 mDa). CCS tolerance was established at <4.05% from the MARS predicted value and RT at <1.5 min from that calculated from NIST RI (Table S2).

#### 2.5.2 Target and Nontarget Screening in GC-EI-QOrbitrap MS

The software used for data processing was Trace Finder 5.2 (Thermo Scientific, Waltham, MA, USA). For target purposes, the homemade database contained retention time, the target ion’s accurate experimental mass (preferably the molecular ion if observed in the EI spectra), and two confirming ions’ accurate experimental masses. European Commission SANTE guideline was also followed for identification purposes: a positive was confirmed based on a target ion (preferably the molecular ion) and two confirming ions from the EI spectra with mass accuracy ≤5 ppm and an RT tolerance ≤0.1 min.

For nontarget screening, the method was based on previous works with some modifications.^11,13^ Deconvolution Plugin 1.5 for Trace Finder 5.2 allowed peak detection with spectral deconvolution and tentative identification of compounds against the NIST 2023/EPA/NIH EI library (EI spectra of 394,000 compounds and retention index, RI, values of 347,100 compounds). Filters used in the peak deconvolution step were a signal-to-noise ratio (S/N) greater than 5, an ion overlap window of 98%, a mass error of ±5 ppm, and a retention time (Rt) aligning window ±10 s. OMPs in samples were tentatively identified by automated comparison of the deconvoluted mass spectra with those present in the NIST 2023 library, obtaining the search index (SI), high-resolution filtering (HRF), and linear retention index (ΔRI) deviation parameters. SI is derived from a modified cosine of the angle between the experimental and library spectra; from 0 to 999. A value of 700 or higher was considered a good match.^3^ HRF represents the percentage of the deconvoluted spectra that can be explained by the molecular formula from a potential library match. Authors typically establish the cutoff value for this parameter at 80.^11,13^

## 3 Results and Discussion

### 3.1 Screening Workflows Using GC-HRMS

Screening workflows for identifying and confirming CECs in environmental and food safety applications have been greatly enhanced by (GC/LC)-HRMS setups. Different strategies can be established depending on the particularities of the screening approach and type of HRMS acquisitions.^2,3,30^ Regarding GC-HRMS interfaces, unlike LC-HRMS setups, they are not limited only to soft ionization sources. On the one hand, the soft ionization of APCI preserves the molecular ion information, which allows for the screening of exact mass lists. This capability enables the development of screening strategies similar to those developed with LC-HRMS or LC-IMS-HRMS.^21^ On the other hand, the extensive and reproducible fragmentation promoted by EI makes NIST searching a more suitable option for nontarget approaches. This is a key aspect with significant implications for data acquisition and screening processing and must be considered when comparing the performance of GC-APCI-IMS-QTOF MS and GC-EI-QOrbitrap MS for wide-scope screening. Figure 1 illustrates the screening process, from data acquisition to feature identification, using both GC-HRMS technologies. The subsequent sections provide a detailed explanation.

**Figure 1:**
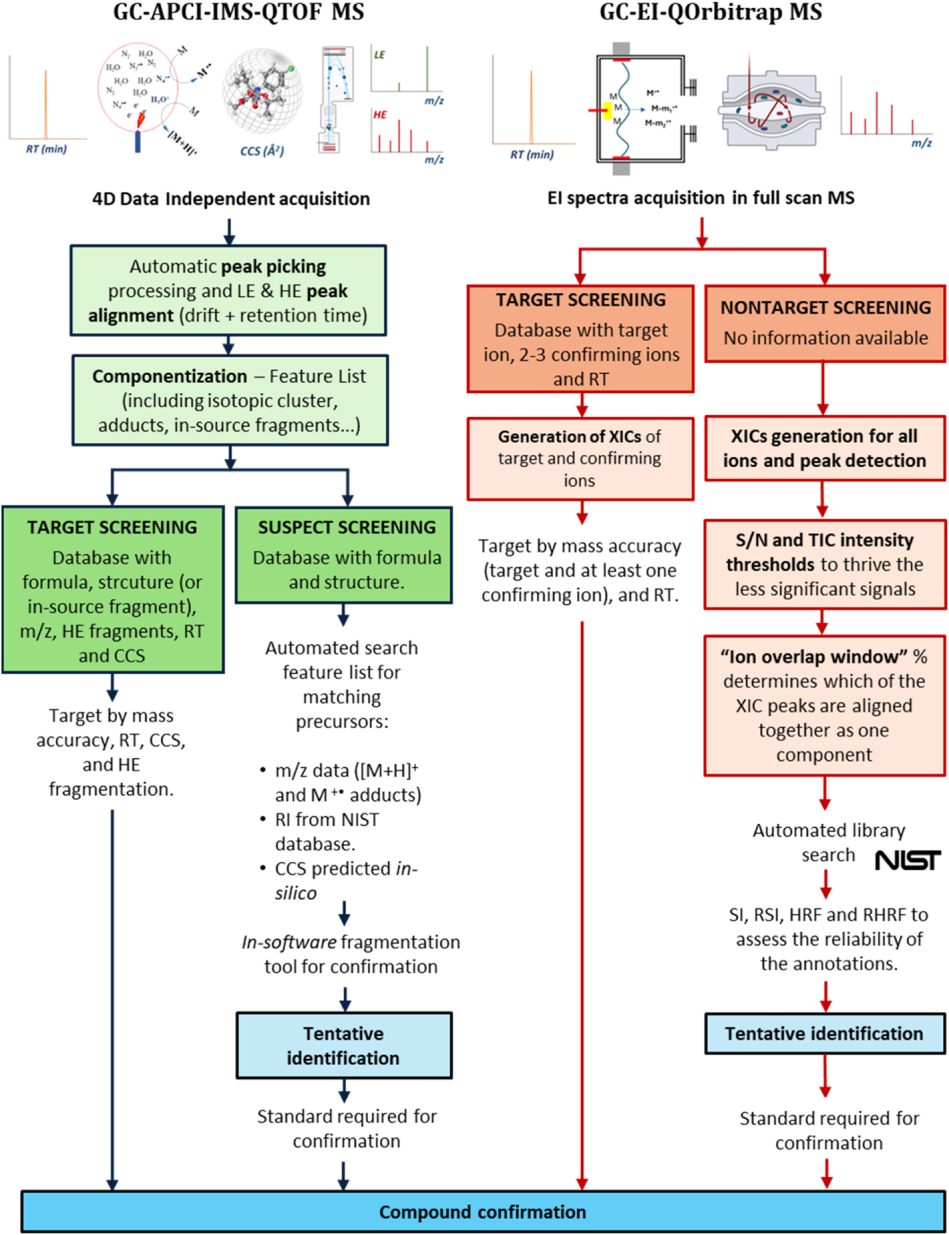
Data acquisition, processing, and feature identification workflow for GC-APCI-IMS-QTOF MS and GC-EI-Orbitrap MS target, suspect, and nontarget screening strategies.

#### 3.1.1 GC-APCI-IMS-QTOF MS Screening Workflow

Figure 1 (on the left) shows the proposed screening workflow when using GC-APCI-QTOF MS. After peak picking, peak alignment, and peak componentization, this hyphenated HRMS technique generates a feature list in which each item contains four dimensions of data: the exact mass of a specific ion in the low collisional energy spectra, retention time, mobility-derived CCS value, and intensity. In addition, a list of ions from the high collision energy spectra collected at the same drift and retention time is attached to the defined component. When following a target approach, the data on each feature can be matched with the information provided by molecular, (de)­protonated, and in-source fragments ions generated from commercially available standards acquired at the same experimental in-house conditions. When this information is inaccessible (i.e., standards not available), it is possible to implement automatable suspect screening strategies since a list containing chemical structures and exact masses can be screened across the feature list. Furthermore, the list can be complemented with the RI available in the NIST and predicted CCS values. Despite not being included in the Figure 1 scheme, it is also possible to work following a nontarget approach. The acquired data can be matched with HRMS and CCS data sets. Unfortunately, most libraries have been acquired using LC-HRMS or LC-IMS-HRMS and are limited to [M + H]^+^ adducts of non-GC amenable species. Furthermore, pipelines for nontarget screening in GC-APCI-HRMS are laborious, time-consuming, and limited to specific functional groups.^31^

#### 3.1.2 GC-EI-QOrbitrap MS Workflow

Figure 1 (on the right) shows the proposed screening workflows for GC-EI-QOrbitrap MS. The extensive fragmentation of the ionization source in this configuration and the acquisition of full MS spectra with high mass accuracy generates unique spectra for each compound in the matrix. When using a target approach, a set of extracted ion chromatograms (XICs) aligned at a specific retention time and corresponding to the most relevant ions observed in the reference standard is used to confirm the presence of the compound in the sample. However, when standards are unavailable, the identification process mainly relies on matching experimental spectra with standardized spectral libraries. The coelution of analytes with matrix interferences is frequent in complex-matrix samples and it affects the match score or may even lead to making a wrong assignment when comparing the spectra with the NIST/EPA/NIH Mass Spectral Library. To minimize this problem, the deconvolution plug-in application for TraceFinder 5.2 takes the “mass spectrum-first” approach.^32^ This strategy generates XICs for all the ions in the chromatogram, and only those aligned in a narrow section around the peak apex are selected as fragments of the same compound. The parameter to modulate the percentage of peak selected is the “ion overlay window,” which makes possible the deconvolution of coeluting chromatographic peaks into multiple components based on their slightly different retention times. Then, the software performs a library search for all the binned components, and additional information based on the spectral and chromatographic match is included to prioritize the best matches.

### 3.2 Target Screening Capabilities: GC-APCI-IMS-QTOF MS versus GC-EI-QOrbitrap MS

A target assessment was conducted to identify contaminants that meet the specific criteria for HRMS as described by EC guidelines (See Sections 2.5.1 and 2.5.2). The results, detailed in Figure 2, provide insights into the efficiency of both GC-HRMS setups for target screening. The GC-APCI-IMS-QTOF MS setup, with mobility separation as an additional parameter, showed a range of 149 to 170 positive assignments. Only a minimal percentage of positives (less than 2%) were discarded due to noncompliance with the mobility requirement, highlighting the reliability of the assignments. Remarkably, many compounds were not detected (19 were not seen in any sample) or did not present matched fragmentation in the HE when using GC-APCI-IMS-QTOF MS configuration. Conversely, the GC-EI-QOrbitrap MS setup demonstrated higher sensitivity, yielding a higher number of positive assignments, ranging between 185 and 195. Table S3 provides a detailed list of the compounds fulfilling target criteria for both systems, including cases where assignments were affected by factors such as mobility and mass accuracy deviations or unmatched fragmentation.

**Figure 2:**
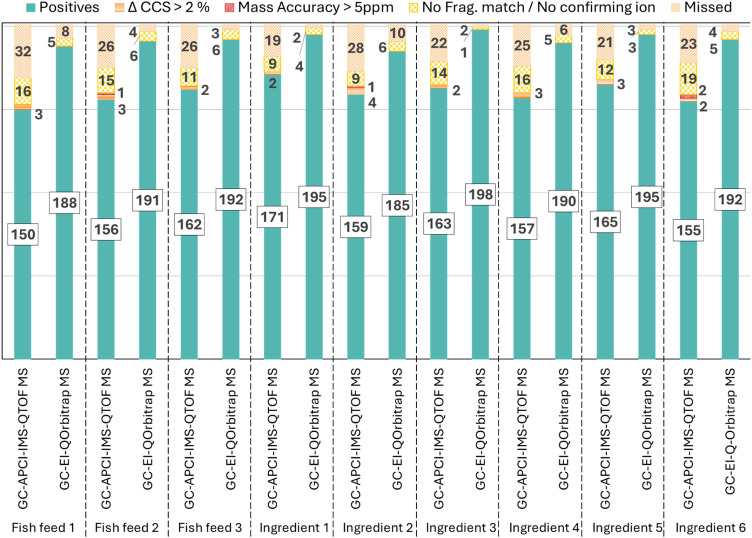
Assessment of SANTE criteria for positive assignments over 201 standards spiked in nine actual samples of feed fish ingredients using two different GC-HRMS configurations—positive assignments in blue with mass accuracy deviations <5 ppm. For GC-APCI-IMS-QTOF MS, molecular/protonated ion and at least one production matched in the homemade database, and empirical CCS < 2% deviated from the standard. For GC-EI-QOrbitrap MS, target and at least a confirming ion.

Two factors can compromise sensitivity in GC-APCI-IMS-QTOF MS: ionization and ion transmission. Regarding ionization, the current findings appear counterintuitive, as the APCI ionization source can enhance sensitivity compared to EI, where extensive fragmentation distributes the signal across numerous fragment ions.^1,6^ However, the ionization yield in APCI can be influenced by side reactions occurring in the ionization chamber, which are affected by the surrounding environment and, consequently, the sensitivity.^33^ This study promoted proton-transfer and charge-transfer reactions under wet and dry chamber conditions, respectively (See Section 2.4.1.). Unfortunately, the open-source chamber design of most available GC-APCI sources, including the one used in this work, allows uncontrolled gas exchange with the surrounding environment, leading to potential fluctuations in humidity levels within the source.^33^

Ion transmission is another critical factor, with the mobility cell potentially acting as a restrictor. While ion mobility improves sensitivity in LC-HRMS systems by removing coeluting interferents and enhancing the S/N ratio,^34^ this advantage may be less pronounced in GC systems, where noise and background levels are inherently lower than in LC. Supporting Information (Figures S1–S5) provides examples of GC-APCI-IMS-QTOF MS acquisitions conducted with the mobility cell activated and deactivated. These comparisons prove that, on the same instrument and under identical conditions, the use of the mobility cell can reduce the signal by a factor of 3 to ten, consequently decreasing the number of detectable fragments.

### 3.3 Suspect Screening Workflows in GC-APCI-IMS-QTOF MS Analysis

#### 3.3.1 Suspect Screening in GC-APCI-IMS-QTOF MS: Tentative Identifications and False Annotations

Automated suspect screening workflows enable rapid monitoring of large data sets of contaminants of interest and ensure consistency in interpreting the results. However, automated detection can lead to misidentifications, requiring careful manual evaluation of results. Hence, applying restrictive parameters to minimize false annotations and reduce the processing time is interesting. While some suspect lists now include additional structural data,^3^ the only information available is often chemical structures and exact masses. In this context, introducing in-silico prediction tools may be helpful in the prioritization strategy for identification. Integrating suspect screening workflows in GC-APCI-IMS-QTOF MS analysis presents notable benefits for enhancing identification performance. Unlike LC-HRMS suspect strategies, where RT is unavailable or has to be predicted through computational tools,^29^ NIST provides RT-derived system-independent Kovats retention indexes (RI) for a wide range of chromatographic columns. Additionally, the mobility-derived CCS values can be obtained from in-silico prediction tools, offering a high degree of orthogonality with other molecular descriptors and potentially serving as a helpful identification parameter.^35^

Figure 3A illustrates the distribution of tentative identifications and false annotations under different restrictive criteria using GC-APCI-IMS-QTOF MS. The number of features considered in the analysis represents the sum of componentized peaks obtained from analyses in wet and dry conditions. As an example of a component assigned as a tentative identification, Figure 3B shows the low and high energy spectra of the insecticide O-ethyl O-p-nitrophenyl phenylphosphonothionate (EPN), the MS, RT, and CCS error values, as well as the fragments proposed by the in-silico fragmentation tool, their mass errors and their XICs aligned with the (de)­protonated peak.

**Figure 3:**
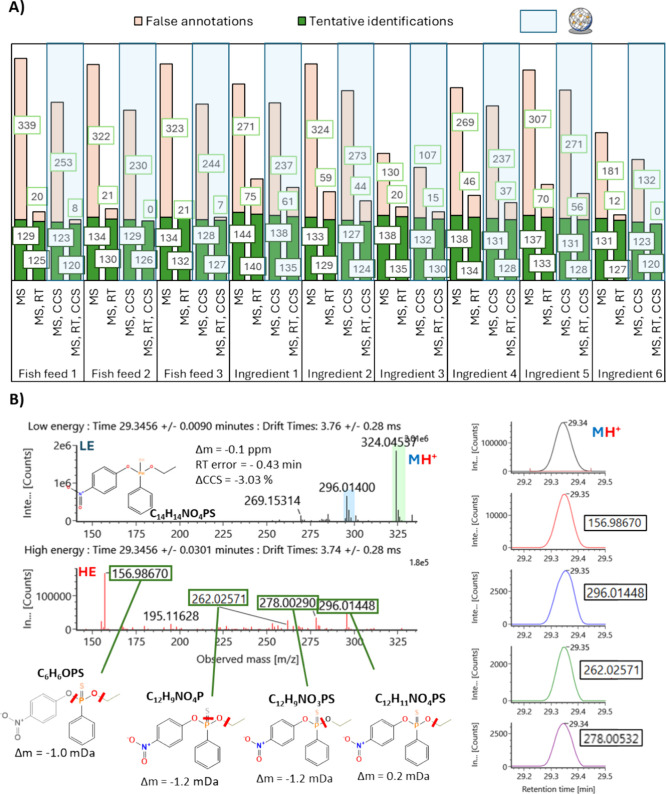
(A) Effect of identification criteria on the number of tentative identifications and false annotations over 201 standards spiked in feed fish samples analyzed with GC-APCI-IMS-QTOF MS through a suspect screening workflow. MS: accurate mass deviation (precursor ions <5 ppm and product ions <2mDa), RT: empirical retention time <1.5 min (from the calculated from KI NIST), CCS: empirical CCS < 4.05% deviated from prediction. (B. Left) Drift time aligned LE and HE spectra of EPN in a spiked sample, green squares indicate fragment ions proposed in-silico. (B. Right) XICs of the protonated ion in LE and proposed fragment ions in HE.

Assessing the impact of MS alone resulted in the highest number of tentative identifications and false annotations across all fish feeds and ingredients. Including RT criteria improved identification performance by reducing false annotations by 85 ± 8%, though it marginally reduced tentative identifications. This reduction mainly affected compounds without a KI in NIST (Table S2) and three compounds with RT errors exceeding 1.5 min: terbumeton-desethyl (−1.66 min), ioxynil octonate (−2.35 min), and fenpropimorph (−3.62 min). By establishing a CCS along with the RT criterion, the number of false annotations was reduced by 91 ± 8% compared to applying only the MS criterion. The total number of tentative positives was also somewhat affected by excluding compounds based on RT and CCS criteria. Specifically, the total number of tentative identifications was reduced by 5 to 8 compounds per sample. Among the compounds outside the CCS deviation threshold were 2 PAHs (benzo­(c)­fluorene (+H) and benzo­[j]­fluoranthene (+H)), 6 pesticides (endosulfan sulfate (+•), fenpropimorph (+H), iprodione (+H), isodrin (+•), metconazole (+H), tebuconazole (+H)), and two emerging compounds (galaxolide (+H) and muskone (+H)), with a maximum deviation lower than 6%. Although the RT tolerance selected is broad for typical screening purposes, it has been demonstrated to be the most influential parameter in reducing the number of false annotations and, consequently, decreasing processing time. Conversely, while CCS contributes to minimizing false annotations, its impact is less significant and affects the number of tentative identifications. The following subsections will discuss the reliability of the tentative assignments, the causes of false negatives compared to target screening, and the suitability of the MARS model for predicting CCS in GC-amenable compounds.

#### 3.3.2 Reliability of the Tentative Assignments

The majority of tentative assignments from the suspect screening were confirmed as positive identifications in the target analysis. Mass accuracy of molecular ions, fragment ions, and retention time derived from NIST RI have proven to be effective parameters for compound annotation. However, in 2.5% of cases, tentative assignments from the suspect screening could not be confirmed as positive in the target analysis due to insufficient fragmentation data. This occurs when the analyte’s fragment ions are too low in abundance to be detected in the high-energy (HE) spectrum, which is instead dominated by ions from coeluting matrix contaminants. Consequently, the in-software fragmentation prediction tool mistakenly identifies these contaminant ions as analyte fragments. For example, Figure S6A illustrates low and high energy spectra of buprofezin in solvent and spiked samples. The two spectra exhibit different ion patterns, with no common ions between them. Additionally, the low-energy spectrum of the fortified sample shows the presence of an interfering matrix coeluent, indicating that the ions in the drift-aligned HE spectrum are from this unknown species. The in-silico fragmentation tool justifies eight ions as buprofezin fragments. However, many of these proposed fragmentations are chemically implausible, such as the loss of a methyl group by C–C bond cleavage in the m/z 247.16834 peak (Figure S6B).

#### 3.3.3 Unveiling False Negatives in Automated Suspect Screening

When comparing the true positive identifications obtained through the target analysis (Figure 2) and the tentative assignments from the suspect screening (Figure 3A), it is observed that the latter approach could not identify 17% of the compounds. Figure 4A outlines the reasons for missed identifications and their significance in the data set. Two primary factors contribute to false negatives: the absence of molecular or (de)­protonated ions due to excessive fragmentation within the ion source (53%) and the lack of explained fragment ions within the HE spectra by the in-silico fragmentation tool (47%). Despite the soft ionization produced by APCI, certain species are susceptible to fragmentation in the source, leading to a marked reduction in the abundance of molecular and (de)­protonated ions. Consequently, these signals may fail to surpass the intensity threshold required for detection in suspect screening. For example, Figure S7 illustrates two low-energy spectra corresponding to methoxychlor (C_16_H_15_Cl_3_O_2_), with and without drift time filtering by ion mobility. In both cases, the methoxychlor’s molecular and/or (de)­protonated ion is absent, but the in-source fragment corresponding to [C_9_H_8_Cl_3_O_2_]^+^ is very abundant. The absence of assigned fragment ions within HE spectra is particularly prevalent in structures composed of aromatic rings such as PAHs, and organophosphate compounds such as tributyl phosphate, tripropyl phosphate, and cadusafos. This issue arises mainly due to the limitations of computational tools in explaining rearrangements in fragmentation pathways for such compounds. For example, Figure S8 shows the HE spectra of anthracene (C_14_H_10_) in spiked ingredient 5 and solvent. The two highlighted fragments ([C_12_H_8_]^+•^ and [C_9_H_7_]^+^), whose structures are proposed in Figure S8, were not justified by the in-silico fragmentation tool. In most PAHs where this lack of structure explanation occurs, CCS threshold was also not fulfilled, with deviations reaching 7%.

**Figure 4:**
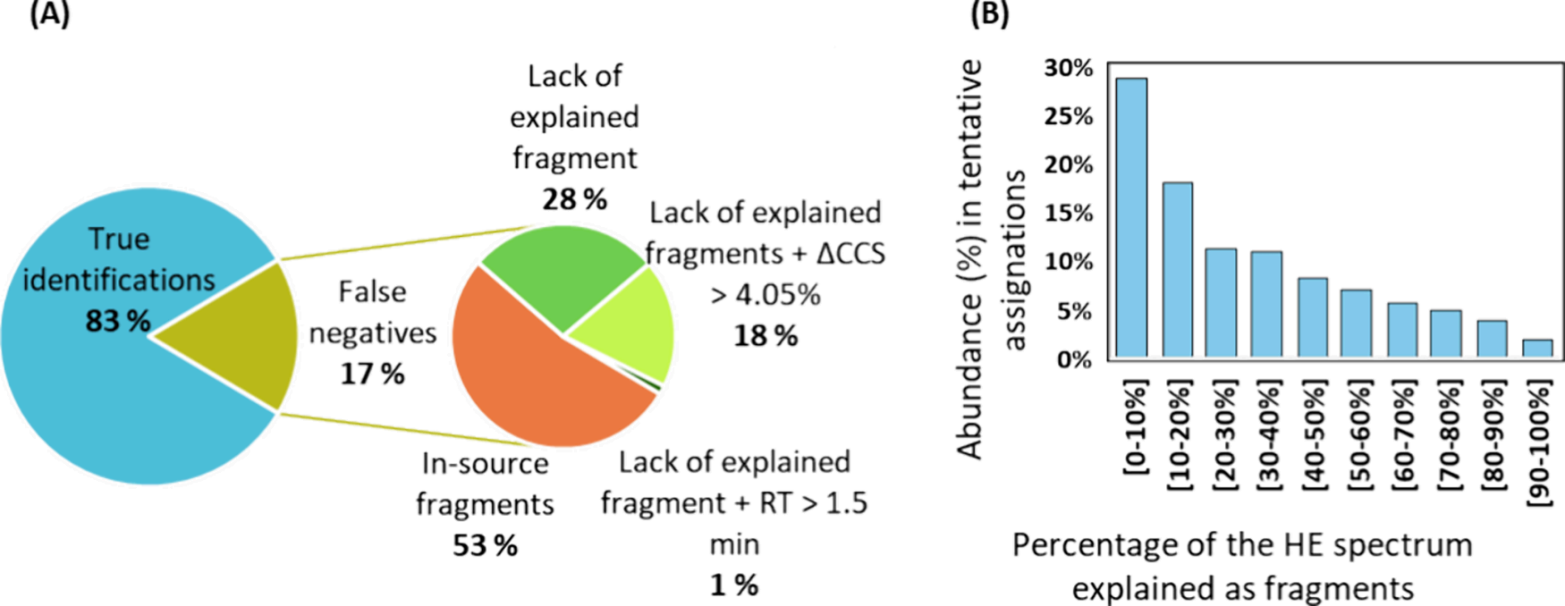
(A) Pie chart showing the sources of false negatives in the suspect screening approach in GC-APCI-IMS QTOF MS when applying MS criteria (precursor ions <5 ppm and product ions <2mDa). (B) Percentage of fragment ions in HE spectra explained as structural fragments *in silico* in the tentative assignments from the suspect screening approach.

#### 3.3.4 Is the In-Silico Interpretation of HDMS^E^ Spectra Reliable for Automated Screening Strategies

Proper assessment of MS/MS spectra is indispensable for ensuring the accuracy of screening strategies. The quantity and quality of fragment ions in HE spectra are crucial for reliable compound identification. Parameters such as the number of ions, their significance in the overall spectrum, and their structural relevance must be carefully considered in screening workflows. The previous sections discussed the limitations of in-silico fragmentation tools based on combinatorial fragmentation. First, nonanalyte ions may be assigned as structural fragments in ion-rich spectra from coeluting matrix interferents. Second, some fragmentation pathways, such as rearrangements, cannot be explained by in-silico tools, which typically only account for simple cleavages.

Alygizakis et al. emphasized the importance of fragmentation score in their automatic prioritization scheme for identifying emerging contaminants. This score, which considers the number of unique fragments detected relative to the total number of fragments in the empirical spectrum or the total number of ions in the spectrum when using *in-silico* tools, highlights the limitations of current methodologies.^36^ Data-independent acquisition can be more affected by matrix interferences, yet it offers the potential for using additional separation dimensions, such as ion mobility, to improve the quality of DIA spectra. Figure 4B evaluates the percentage of explained product ions across all drift-aligned HE spectra for tentative identifications generated through the suspect screening approach. A concerning trend is revealed: in-silico fragmentation tools fail to explain even 30% of the HE spectra in more than half of the cases (28% + 17%+ 10% = 55%). Using the identification scoring system proposed by Alygizakis et al., most assignments would not attain Level 4 status.^36^ While the limitations of fragment prediction tools have been underscored previously, it is essential to acknowledge that, despite the additional separation capability provided by ion mobility, equipment with low mobility resolution—such as the current TWIMS used in this work—cannot entirely remove interferences in DIA. Therefore, enhancing the understanding and mitigation of these limitations is critical for advancing the reliability and effectiveness of automated screening strategies. It is noteworthy that other variations of TWIMS-based technologies, such as Structures for Lossless Ion Manipulations (SLIM)^37^ or cyclic IMS,^38^ can considerably enhance the separation power of ion mobility.

#### 3.3.5 CCS Prediction Accuracy for GC-Amenable Compounds

The accuracy of predicting CCS for GC-amenable compounds was assessed by comparing the predicted values from the CCS_H_ MARS prediction model with those reported for [M + H]^+^ and M^+•^ by Izquierdo-Sandoval et al.^20^ It is noteworthy that this model was optimized initially and trained for [M + H]^+^ adducts from chemicals amenable to LC and ionized by electrospray ionization (ESI+).^29^ Its potential implementation in APCI-based GC-IMS systems has not been explored until now. Figure S9 shows the correlation between empirical and predicted data (top) and violin plots illustrating the distribution of deviations (bottom) for both ion species, molecular and (de)­protonated. Both deviation distributions exhibited a positive bias, with average deviations of 0.72% for [M + H]+ and 1.76% for M^+•^. For [M + H]^+^, 95% fell within the range of −3.75% to 6.34%, while for M^+•^, the range exhibited a slightly positive bias from −2.32% to 7.43%. As noted in the previous section, the PAH chemical family showed significant deviation from the model threshold. Except for naphthalene, PAHs exhibited deviations of 4.17–7.26% for [M + H]^+^ and 4.58–9.67% for M^+•^. This discrepancy impacts the results of the suspect screening approach by potentially increasing the rate of false negatives, as the CCS deviations for PAHs and other species fall outside the acceptable range, thereby reducing the identification performance.

To address these challenges, a potential solution would be to develop and validate a CCS_H_ prediction model based on a comprehensive data set of GC-amenable compounds. By incorporating PAHs and other relevant GC-amenable contaminant families into the training data set, the model could better account for the unique structural features and ionization behaviors of these molecules, leading to improved predictive performance and reduced deviations. Furthermore, establishing a CCS prediction model specifically for molecular ions (M+•) is crucial, as many contaminants exhibit substantial differences between the experimental CCS values of the two ionization forms.^20^

### 3.4 Nontarget Screening Workflows in GC-QOrbitrap MS

#### 3.4.1 Nontarget Screening Workflows in GC-QOrbitrap MS: Reducing the Number of Potential Annotations without Sacrificing True Identifications

Implementing screening strategies using a predefined list of molecular formulas is complex due to the high fragmentation of EI sources. However, the robustness of EI spectra and the existence of the NIST library play a crucial role in facilitating the development of nontarget strategies, allowing the detection of a wide range of chemical families without the need to preselect a list of suspects. The analysis and processing of the nine spiked matrices using the “mass spectrum-first” approach applied in the GC-EI-QOrbitrap MS (see Section 3.1.2) generated between 1,000 and 3,000 deconvoluted and NIST-annotated spectra. A significant challenge when using these strategies is selecting the optimal parameters to achieve the highest number of reliable identifications, reducing the total number of annotated compounds to be reviewed. To this purpose, this section examined the influence of different matching spectral parameters (SI > 700 and HRF > 80%) and Kovats RI (ΔRI < 5%) on prioritizing tentative positives. Figure 5A illustrates the number of true identifications and the remaining annotated compounds in the spiked samples under the application of the combination of these different criteria.

**Figure 5:**
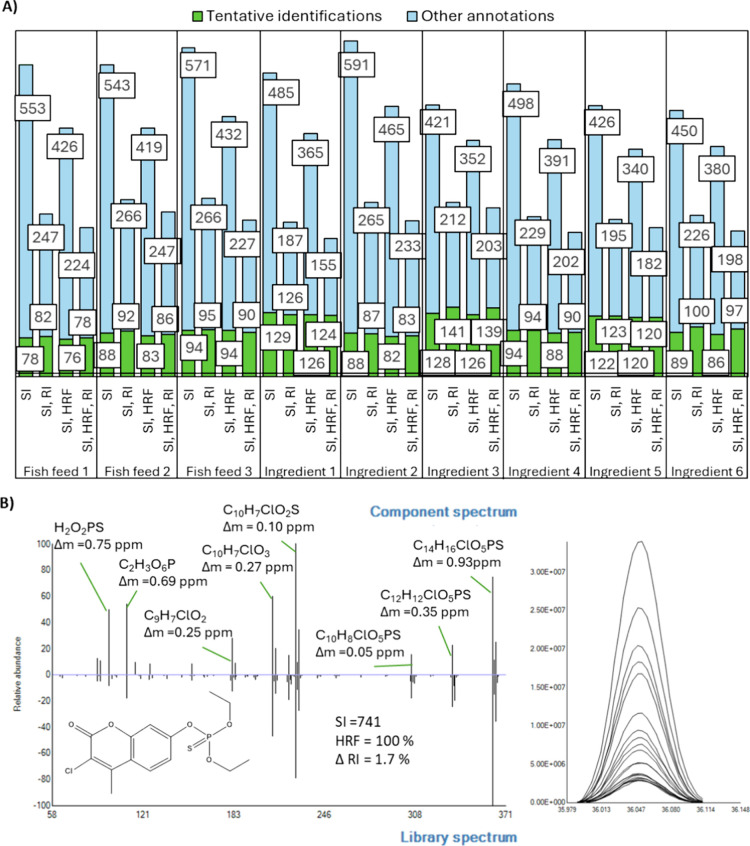
(A) Effect of identification criteria on the number of true identifications and other annotations over 201 standards spiked in feed fish samples. SI < 700, HRF < 80%, RI < 5%. (B. Right) Deconvoluted EI spectrum acquired with GC-QOrbitrap MS in a spiked sample (top) library NIST spectrum (bottom) for coumaphos. Peaks used for calculating the HRF factor are marked with a green line. (B. Left) XICs of the ions conforming to the deconvoluted EI spectrum. spectrum (bottom) for coumaphos. Peaks used for calculating the HRF factor are marked with a green line.

Applying the SI filter substantially reduced the number of annotated peaks across the samples (556 ± 56), with the number of positive identifications ranging between 41% and 66% of the total positives found in the target analysis. Adding the RI filter considerably reduced the number of annotated peaks to 294 ± 16 and slightly increased the number of positive identifications (44% – 72%). Additionally, implementing the HRF cutoff increased the level of restriction, further reducing the total number of compounds but only marginally affecting the total number of positives by a maximum of six compounds in ingredients 2 and 4. The most restrictive scenario occurs with the combination of SI, HRF (%), and ΔRI (%), which resulted in an averaged annotated peak of 269 ± 20 and an average of positives of 91 ± 18 (41% to 70%) of the total positives found in target. As an example of a component assigned as a tentative identification fulfilling all the established criteria, Figure 5B shows the match of deconvoluted EI spectra for coumaphos to the library NIST spectrum in the spiked Ingredient 5.

Unlike the suspect screening performed in GC-APCI-IMS QTOF MS, the application of more selective criteria does not necessarily imply a reduction in the number of positives since the RI and the HRF could help to prioritize an analyte over other potential matches with a better SI. For example, Figure S10 shows the deconvoluted spectrum of chlorpropham in ingredient 1 with a good library match (SI: 862, HRF: 100%, and ΔRI: 0.5%). However, if RI is not considered as a criterion, this compound would be annotated as a species with NIST ID 138367 whose RI is absent (SI: 889 and HRF: 100%). In contrast, Figure S11 shows an example of a discarded positive of hexachlorobenzene by the application of HRF criteria. The presence of coeluting matrix interfering peaks penalized the percentage of the spectrum that could be justified by the molecular formula of hexachlorobenzene but did not modify the spectrum distribution enough to affect the SI. In this case, reverse HRF could be used as additional information to make the assignment more reliable.

#### 3.4.2 Unveiling False Negatives in Nontarget Screening

The number of spiked compounds correctly identified in the nontarget workflow accounted for only 52.55% of the true identifications obtained through the target approach. To understand the limitations that caused the nontarget method to miss the remaining 47.45% of compounds, the sources of these false negatives are analyzed in Figure 6. The primary reason for these missed identifications (40.42% of the false negatives) was the inability of the nontarget pipeline to deconvolute a distinct and consistent spectrum corresponding to the fragment ions of the spiked contaminant (“undetected compounds”). In 33.46% of the false negatives, although the peak was deconvoluted, the corresponding spectrum did not show enough similarity to the library, resulting in SI values below 700. For example, as shown in Figure S12, indoxacarb had a low SI value (452) because several key peaks from the library spectrum were absent in the component spectrum. However, HRF results in a high value (98%) since a formula derived indoxacarb molecular formula can be assigned to almost all the peaks with minimal mass error. Other causes of false negatives included the absence of an RI value in the NIST library (9.65%), failure to meet the HRF criteria (6.35%), and cases where both HRF and RI criteria were not satisfied (0.61%). Lastly, in 9.52% of the false negatives, even though the SI, HRF, and RI thresholds were met, other compounds in the library had higher matching scores. Figure S13 illustrates this situation with tonalid, which was spiked into the sample, but a higher match score was obtained for 7-Acetyl-6-ethyl-1,1,4,4-tetramethyltetralin (NIST ID 2260024). Similar cases were observed with some PAHs and organochlorine insecticides such as DDD, DDT, DDE, and polychlorinated biphenyls.

**Figure 6:**
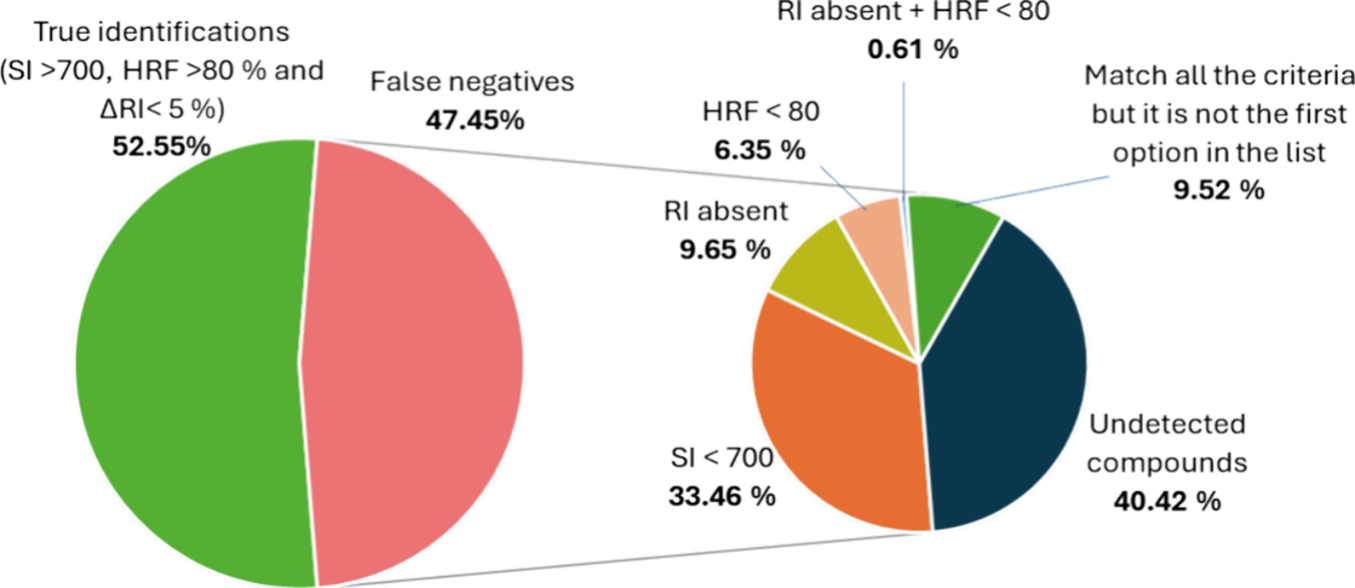
Pie chart showing the sources of false negatives in the nontarget approach in GC-QOrbitrap MS applying the following criteria: SI < 700, HRF < 80%, and ΔRI < 5%.

### 3.5 Confidence Levels in GC-HRMS

The previous sections examined the capabilities of both GC-HRMS configurations in prioritizing tentative positive GC-amenable contaminants in complex matrices. While the GC-APCI-IMS-QTOF MS system demonstrated lower sensitivity compared to GC-EI-QOrbitrap MS in target approaches, its suspect screening approach yielded a higher number of true annotations than the nontarget screening approach performed with GC-EI-QOrbitrap MS. It is important to remark that this comparison is based on a controlled case study involving a known list of spiked compounds in complex matrices, in real-world applications, where reference standards for confirmation may be unavailable, the confidence of annotated candidates must be considered carefully assessed for prioritization purposes. The five-level classification system proposed by Schymanski et al.^23^ is the most widely used scoring system for environmental applications, and its effectiveness has been demonstrated in suspect screening with GC-APCI-QTOF MS.^32,39^

Identifications obtained through the automated workflow proposed for suspect screening with GC-APCI-IMS-QTOF MS can be categorized as either unambiguous structures proposed by diagnostic evidence (Level 2b) or tentative candidates without enough information to distinguish them from other structures (Level 3).^22^ The assessment of this approach with spiked matrices showed that the combination of MS, RT, and CCS criteria effectively eliminates the vast majority of false annotations, leaving mostly unique structures. However, several limitations must be considered. First, although it allows obtaining cleaner mass spectra, ion mobility using the technology of TWIMS of the current work has not shown sufficient resolving power to eliminate matrix coeluents in all cases, leading to HE spectra potentially being populated by ions from matrix-coeluting interferents. Second, in-silico fragmentation tools can generate highly unlikely proposed fragments of the analyte that actually come from an interferent. Although in-silico fragmentation tools can be used for prioritization, it is recommended to ensure the reliability of the fragmentation pattern. In this context, alternative in-silico fragmentation prediction software, such as SIRIUS^40^ or MetFrag,^41^ could enhance fragment prediction accuracy. One advantage of using APCI as an ionization source is that some spectra coming from species ionized as [M + H]^+^ can be found in open spectral libraries such as MassBank (MoNA)^42,43^ or Global Natural Products Social Molecular Networking (GNPS).^44,45^

In the case of the nontarget approach using GC-QOrbitrap MS, comparing experimental and empirical spectra available in the NIST database provides a higher level of confidence (Level 2a) for candidate identification. The standardized fragmentation patterns provided by the NIST library facilitate reliable spectral matching, which is essential for accurate identification. Combining SI and HRF provides a robust criterion for assessing the spectra matching of NIST candidates. The SI score is based on the dot product metric between two spectra in nominal masses while HRF uses the exact masses of the fragments to calculate the percentage of the spectra that can be matched by a given formula of the NIST candidate. Additionally, filtering candidates by RI further minimizes the risk of misidentifications, although care must be taken with isomers that may share similar spectra and RI values, as multiple candidates could fit a tentative identification. Special consideration should be given to these limitations in both GC-HRMS configurations to ensure a thorough assessment of all potential candidates. Furthermore, the NIST library may not include new and emerging substances that have not yet been characterized or added to the database, which can hinder the identification of novel contaminants of increasing relevance in environmental monitoring. A significant limitation encountered in the nontargeted workflow with GC-EI-QOrbitrap MS was the inability to effectively deconvolute independent spectra for a considerable number of spiked compounds. To address this challenge, alternative commercial software (e.g., Compound Discoverer) or open-access platforms (e.g., MS-DIAL^46^ or MZ-mine^47^), may serve as complementary tools for the deconvolution of GC-HRMS data and the subsequent annotation of compounds.^3.^

## Supplementary Material



## Notes

Parameters used for screening in Waters Unifi software; suspect list for GC-APCI-IMS-HRMS; detailed target analysis of spiked samples using both GC-HRMS techniques; examples of GC-APCI-IMS-QTOF MS acquisitions with mobility cell activated and deactivated; correlation and statistical analysis between experimental and predicted CCS values; illustrative examples of potential sources of false assignations and false negatives in both GC-HRMS techniques (PDF)
