# Supplementary material for: Approaches for GC-HRMS Screening of Organic Microcontaminants: GC-APCI-IMS-QTOF versus GC-EI-QOrbitrap
Source: Environ Sci Technol. 2025 Jan 31;59(5):2436–48. doi: 10.1021/acs.est.4c11032 (PMC12129252; doi:10.1021/acs.est.4c11032)
Supplement: Supplementary file 1 [file es4c11032_si_001.pdf]

Supporting Information

Approaches for GC-HRMS screening of organic microcontaminants: GC-APCI-IMS-QTOF versus GC-EI-Q-Orbitrap

David Izquierdo-Sandoval[1], Juan Vicente Sancho[1], Félix Hernández[1], Tania Portoles[1]*

[1] Environmental and Public Health Analytical Chemistry, Research Institute for Pesticides and Water (IUPA), University Jaume I, Av. Sos Baynat S/N, 12071, Castellón de la Plana, Spain.

*Dr. Tania Portolés (ORCID: 0000-0002-8422-0822) E-mail. tportole@uji.es

Phone: +34-964 387337 Fax: +34-964 387368

Summary: 37 pages, 13 figures, 3 tables.

TABLE OF CONTENTS:

Table S1. Parameters used for screening in Waters Unifi software. S4

Table S2. Suspect list for GC-APCI-IMS-HRMS including the compound name, molecular formula, the family to which it belongs, experimental RI for semi-standard non-polar columns (NIST1), retention time calculated from RI NIST values, and CCS predicted by MARS. S7

Table S3. Target assessment of fortified samples in spiked feed. For GC-APCI-VION-QTOF MS, positive assignments (▪), CCS > 2% (CCS), no fragmentation matched with the databaase (❶), and mass accuracy > 5 ppm (☒) . For GC-EI-QOrbitrap MS, positive assignments (▫), and no confirming ion detected (①)............ S13

Figure S1. (Left) Narrow window-extracted ion chromatogram (nw-XIC) of phosmet at 10 ng · mL^{-1} in hexane acquired in GC-APCI-IMS-QTOF MS in MS^E mode. (Right) Narrow window-extracted ion chromatogram (nw-XIC) of phosmet at 10 ng • mL-1 in hexane acquired in GC-APCI-IMS-QTOF MS in $HDMS^E$ mode............ S26

Figure S2. (Left) Narrow window-extracted ion chromatogram (nw-XIC) of pendimethalin at 10 ng · mL^{-1} in hexane acquired in GC-APCI-IMS-QTOF MS in MS^E mode. (Right) Narrow window-extracted ion chromatogram (nw-XIC) and drift time aligned nw-XIC of pendimethalin at 10 ng · mL^{-1} in hexane acquired in GC-APCI-IMS-QTOF MS in $HDMS^E$ mode. S27

Figure S3. (Left) Narrow window-extracted ion chromatogram (nw-XIC) of metalaxyl at 10 ng · mL^{-1} in hexane acquired in GC-APCI-IMS-QTOF MS in MS^E mode. (Right) Narrow window-extracted ion chromatogram (nw-XIC) and drift time aligned nw-XIC of metalaxyl at 10 ng · mL^{-1} in hexane acquired in GC-APCI-IMS-QTOF MS in $HDMS^E$ mode. S28

Figure S4. (Left) Narrow window-extracted ion chromatogram (nw-XIC) of EPN at 10 ng · mL^{-1} in hexane acquired in GC-APCI-IMS-QTOF MS in MS^E mode. (Right) Narrow window-extracted ion chromatogram (nw-XIC) and drift time aligned nw-XIC of EPN at 10 ng · mL^{-1} in hexane acquired in GC-APCI-IMS-QTOF MS in $HDMS^E$ mode............ S29

Figure S5. (Left) Narrow window-extracted ion chromatogram (nw-XIC) of firponil at 10 ng · mL^{-1} in hexane acquired in GC-APCI-IMS-QTOF MS in MS^E mode. (Right) Narrow window-extracted ion chromatogram (nw-XIC) and drift time aligned nw-XIC of fipronil at 10 ng · mL^{-1} in hexane acquired in GC-APCI-IMS-QTOF MS in $HDMS^E$ mode............ S30

Figure S6. A) Low- and high-energy spectra ($HDMS^E$) of buprofezin in GC-APCI-IMS QTOF MS in solvent (left) and spiked sample, ingredient 5 (right), showing analyte fragments (green rectangle) and the false assignations of the in silico fragmentation tool (red rectangle). B) False assignations structures and mass errors............ S31

Figure S7. Drift time-filtered LE and LE spectra of methoxychlor in the spiked sample (fish feed 1), red cross indicates the absence of the molecular/(de)protonated ion of the analyte. S32

Figure S8. LE and HE spectra of anthracene in a spiked sample (ingredient 5) and HE spectra in the solvent, green squares indicate fragment ions from the analyte proposed structures for the fragment ions are displayed. S32

Figure S9. Top: Comparison of experimental and predicted (A) CCS for protonated using the CCS_H model and (B) CCS for molecular ions using the CCS_H model. Bottom: Violin plot of deviations between experimental and predicted data for CCS for protonated molecules using the CCS_H model. Dashed green and orange lines encompass 95% of the deviations for $[M+H]^+$ and $M^{+\bullet}$, respectively. The dashed red line displays the threshold established for the CCS_H prediction model (95% confidence interval). S33

Figure S10. Experimental EI spectrum acquired with GC-Q-Orbitrap MS (A), NIST spectrum for NIST ID 138367 (B), and chlorpropham (C). Peaks used for calculating the HRF factor are marked with a green line S34

Figure S11. Comparison of experimental EI spectrum acquired with GC-Q-Orbirap MS (top) and NIST spectrum (bottom) for hexachlorobenzene. Peaks used for calculating the HRF factor are marked with a green line. The red box indicates the peaks that penalize the HRF factor. S35

Figure S12. Comparison of experimental EI spectrum acquired with GC-Q-Orbitrap MS (top) and NIST spectrum (bottom) for indoxarb. Peaks used for calculating the HRF factor are marked with a green line. The peaks in red are fragments with Cl that do not present the correct isotopic pattern. S36

Figure S13. Experimental EI spectrum acquired with GC-Q-Orbitrap MS (A), NIST spectrum for NIST ID 260024 (B), and tonalid (C). Peaks used for calculating the HRF factor are marked with a green line. S37

Table S1. Parameters used for screening in Waters Unifi software.

**Peak processing settings**	
Find 2D peaks	
Chromatographic peak width	Automatic peak width
Peak detection threshold	Automatic detection
Peak integration liftoff	0
Peak integration touchdown	0.5
Peak rejection min. height	0
Peak rejection min area	100
Max # of allowed peaks per chromatogram:	1000
Calculate detector noise and drift	Activated
RT range (min)	0.1 - 0.5
Segment width	0.25
Smooth algorithm	Mean
Half width	2
Iterations	2
Background subtract	Automatic
Find 4D peaks	
RT range (min):	4.0 - 47.0
MS resolution	Automatic
Peak widths:	Automatic
Apply lock mass correction	Yes
Mass range (high energy)	Automatic
Mass range (low energy)	Automatic
Intensity threshold (high energy)	20 counts
Intensity threshold (low energy)	20 counts
Background filter	High
Maximum number of peaks to keep per channel	200000
4D isotope clustering	
Fraction of chromatographic peak width to apply during cluster creation	0.15
Fraction of chromatographic peak width to apply during high-to-low energy association	0.5
Fraction of drift peak width to apply during cluster creation	0.5
Fraction of drift peak width to apply during high-to-low energy association	0.5
Intensity threshold to apply during high to low energy associations	50
Maximum considered charge for cluster	1
Maximum number of isotopes per cluster	7
Minimum allowed monoisotopic/ largest isotope intensity ratio	0.2
Allow wider chromatographic tolerance for saturated data	Yes
**Targeted screen settings**	
Target by retention time	
Enable screen by retention time	No
Target by mass	
Generate predicted fragments from structure	Yes
Fragment match tolerance	2 mDa
Allow scores below	10

Table S1. Parameters used for screening in Waters Unifi software.

Keep all fragments	Yes
Look for in-source fragments	Yes
Ion ratio tolerance	10%
Extract drift mass-specific chromatograms	Yes
Tolerance	10 mDa
Exclude unobserved targets	Yes
If more than one target component is assigned to candidate then display	All matching targets
Maximum number of targets with the same retention time to keep	10
Minimum relative intensity threshold for targets at the same *m/z*	0.01
Maximum candidates per sample to use during screening and recovery	50000
Maximum candidates per sample to keep after screening and recovery	10000
Screen by collisional cross section	
Enable screen by collisional cross-section	No
**Discovery settings**	
Enable mass defect search	No
Enable common fragment search	No
Enable common neutral loss search	No
Enable potential isomer finder	No
Enable halogen search	No
**Quantitation settings and sample comparison settings**	Not used
**Analysis specific settings**	
Adducts	+H and -e
Lock mass settings	
Combine width	3 scans
Mass window	0.5 m/z
Reference mass 1	355.0699 m/z
Reference charge 1	1
Reference mass 2	not used
Reference charge 2	not used
Lock drift	not enabled
MS resolution	Automatic
**Analysis specific settings - continued**	
Processing rules	
Enforce minimum area-change for manual integration	No
Chromatographic performance settings	
Enable chromatographic performance/system suitability	Yes
Select on the basis of the calculations (All) or the specific pharmacopeia to apply	All
Calculate chromatographic performance for unidentified peaks	No
Column void time	(blank)

Table S1. Parameters used for screening in Waters Unifi software.

Use a noise result reference to calculate signal to noise	No
Force signal to noise calculations to be based on the European pharmacopeia	No
Noise value	Noise
Chromatographic performance settings - continued	
Measure signal from	Peak height
**Custom fields and limits**	(blank)

Table S2. Suspect list for GC-APCI-IMS-HRMS including the compound name, molecular formula, the family to which it belongs, experimental RI for semi-standard non-polar columns (NIST1), retention time calculated from RI NIST values, and CCS predicted by MARS.

Compound	Formula	Family	LRI NIST	RT_{calc} (min)	CCS_{pred} (Å^2)
DEET	C12H17NO	Insect repellent	1583	13.4	143.83
Cashmeran	C14H22O	Musk	1503	11.6	147.00
Galaxolide	C17H22O	Musk	1837	18.7	160.08
Muskone	C16H30O	Musk	1831	18.6	155.11
Tonalid	C18H26O	Musk	1849	19.0	164.26
Traseolide	C18H26O	Musk	1829	18.6	165.20
TBEP H (Tri(2-butoxyethyl) phosphate)	C18H39O7P	OPFRs	2360	28.2	198.69
TBP H (Tributyl phosphate)	C12H27O4P	OPFRs	1613	14.0	164.8
TCEP H (Tri(2-chloroethyl) phosphate)	C6H12Cl3O4P	OPFRs	1766	17.3	149.28
TCrP H (isomer I) (Triresyl phosphate)	C21H21O4P	OPFRs	2545	31.1	187.01
TCrP H (isomer II) (Tricresyl phosphate)	C21H21O4P	OPFRs	2664	32.8	190.78
TCrP H (isomer III) (Tricresyl phosphate)	C21H21O4P	OPFRs			192.8
TDCP H (Tris(1,3- dichloro-2-propyl) phosphate)	C9H15Cl6O4P	OPFRs	2307	27.3	170.78
TPhP H (Triphenyl phosphate)	C18H15O4P	OPFRs	2366	28.3	175.43
TPrP H (Tripropyl phosphate)	C9H21O4P	OPFRs	1372	8.6	148.68
5-Methylchrysene	C19H14	PAHs	2577	31.6	156.98
Acenaphthene	C12H10	PAHs	1481	11.1	130.20
Acenaphthylene	C12H8	PAHs	1454	10.5	130.01
Anthracene	C14H10	PAHs	1767	17.3	138.72
Benz[a]anthracene	C18H12	PAHs	2445	29.5	154.2
benzo(c)fluorene	C17H12	PAHs	2236	26.1	150.16
Benzo[a]pyrene	C20H12	PAHs	2838	35.3	159.57
Benzo[b]fluoranthene	C20H12	PAHs	2705	33.4	159.88
Benzo[ghi]perylene	C22H12	PAHs	3144	39.3	163.27
Benzo[j]fluoranthene	C20H12	PAHs	2704	33.4	159.45
Chrysene	C18H12	PAHs	2453	29.7	153.9
Dibenz[a,h]anthracene	C22H14	PAHs	3099	38.7	170.07
Fluoranthene	C16H10	PAHs	2062	23.4	143.63
Fluorene	C13H10	PAHs	1587	13.4	136.51
Indeno[1,2,3-cd]pyrene	C22H12	PAHs	3086	38.6	166.37
Naphthalene	C10H8	PAHs	1178	4.7	123.42
Phenanthrene	C14H10	PAHs	1784	17.7	137.39
Pyrene	C16H10	PAHs	2114	24.0	142.36
PCB 101	C12H5Cl5	PCBs	2130	24.3	157.23
PCB 118	C12H5Cl5	PCBs	2279	26.9	158.2

Table S2. Suspect list for GC-APCI-IMS-HRMS including the compound name, molecular formula, the family to which it belongs, experimental RI for semi-standard non-polar columns (NIST1), retention time calculated from RI NIST values, and CCS predicted by MARS.

Compound	Formula	Family	LRI NIST	RT_{calc} (min)	CCS_{pred} ($Å^2$)
PCB 180	C12H3Cl7	PCBs	2473	30.0	165.74
PCB 28	C12H7Cl3	PCBs	1831	18.6	149.37
PCB-138	C12H4Cl6	PCBs	2350	28.0	161.42
PCB-153	C12H4Cl6	PCBs	2325	27.6	161.61
PCB-52	C12H6Cl4O2S	PCBs	1900	20.0	149.44
2-Phenylphenol	C12H10O	Pesticides	1515	11.8	134.47
4,4'-Dichlorobenzophenone	C13H8Cl2O	Pesticides	1979	22.3	152.48
Alachlor	C14H20ClNO2	Pesticides	1903	20.1	156.54
Aldrin	C12H8Cl6O	Pesticides	1998	22.9	159.39
Atrazine	C8H14ClN5	Pesticides	1755	17.1	148.29
Atrazine deisopropyl	C5H8ClN5	Pesticides	1665	15.2	138.3
Atrazine desethyl	C6H10ClN5	Pesticides	1654	14.9	133.25
Azaconazole	C12H11Cl2N3O2	Pesticides	2216	25.8	161.29
Azinphos-ethyl	C12H16N3O3PS2	Pesticides	2556	31.3	176.51
Azinphos-methyl	C10PN3H12S2O3	Pesticides	2525	30.8	122.48
Azoxystrobin	C22H17N3O5	Pesticides	3083	38.5	196.16
Bifenthrin	C23H22ClF3O2	Pesticides	2468	29.9	140.83
Bixafen	C18H12Cl2F3N3O	Pesticides	2891	35.3	187.17
Bromophos	C8H8BrCl2O3PS	Pesticides	2026	23.1	158.35
Bromophos-ethyl	C10H12BrCl2O3PS	Pesticides	2134	24.4	167.48
Bromopropylate	C17H16Br2O3	Pesticides	2478	30.1	155.44
Bromuconazole I	C13H12BrCl2N3O	Pesticides	2487	30.2	165.78
Bromuconazole II	C13H12BrCl2N3O	Pesticides	2487	30.2	165.78
Buprofezin	C16H23N3OS	Pesticides	2205	25.6	175.18
Cadusafos	C10H23O2PS2	Pesticides	1692	15.7	158.32
Captan	C9H7Cl2NO2S	Pesticides	2000	22.9	150.16
Carbofuran	C12H15NO3	Pesticides	1743	16.8	147.11
Carfentrazone ethyl	C15H14Cl2F3N3O3	Pesticides	2327	27.7	185.14
Chinomethionate	C10H6N2OS2	Pesticides	2115	24.0	147.49
Chlorfenapyr	C15H11BrClF3N2O	Pesticides	2222	25.9	175.18
Chlorfenson	C12H8Cl2O3S	Pesticides	2089	23.7	157.36
Chlorfenvinphos	C12H14Cl3O4P	Pesticides	2015	23.0	169.25
Chloropyriphos-methyl	C7H7Cl3NO3PS	Pesticides	1902	20.0	154.65
Chlorothalonil	C8Cl4N2	Pesticides	1829	18.6	140.38
Chlorpropham	C10H12ClNO2	Pesticides	1660	15.0	145.64
Chlorpyrifos-ethyl	C9H11Cl3NO3PS	Pesticides	1997	22.2	165.18
Clodinafop-propargyl	C17H13ClFNO4	Pesticides	2379*	28.5*	181.01
Coumaphos	C14H16ClO5PS	Pesticides	2654	32.7	178.45
Cyanazine	C9H13ClN6	Pesticides	1987	22.5	154.02

Table S2. Suspect list for GC-APCI-IMS-HRMS including the compound name, molecular formula, the family to which it belongs, experimental RI for semi-standard non-polar columns (NIST1), retention time calculated from RI NIST values, and CCS predicted by MARS.

Compound	Formula	Family	LRI NIST	RT_{calc} (min)	CCS_{pred} (Å^2)
Cyanophos	C9H10NO3PS	Pesticides	1776	17.5	149.21
Cyproconazole	C15H18ClN3O	Pesticides	2238	26.2	167.83
Cyprodinil	C14H15N3	Pesticides	2068	23.5	154.36
DDD	C14H10Cl4	Pesticides	2213	25.7	147.65
DDE	C14H8Cl4	Pesticides	2196	25.5	163.34
DDT	C14H9Cl5	Pesticides	2300	27.2	147.65
Deltamethrin	C22H19Br2NO3	Pesticides	3059	38.2	201.32
Diazinone	C12H21N2O3PS	Pesticides	1791	17.8	165.39
Dichlofenthion	C10H13Cl2O3PS	Pesticides	1873	19.5	161.06
Dichloran	C6H4Cl2N2O2	Pesticides	1734	16.6	133.99
Dichlorvos	C4H7Cl2O4P	Pesticides	1258	6.2	136.33
Dicofol	C14H9Cl5O	Pesticides	2467	29.9	152.48
Dicrotophos	C8H16NO5P	Pesticides	1645	14.7	148.28
Dieldrin	C12H8Cl6O	Pesticides	2109	23.9	161.03
Difenoconazole	C19H17Cl2N3O3	Pesticides	3017	37.7	195.42
Diflufenican	C19H11F5N2O2	Pesticides	2397	28.8	184.8
Dimethachlor	C13H18ClNO2	Pesticides	1847	18.9	154.09
Dimethoate	C5H12NO3PS2	Pesticides	1736	16.7	141.99
Dioxathion	C12H26O6P2S4	Pesticides	1742	16.8	153.23
Diphenylamine	C12H11N	Pesticides	1634	14.5	137.5
EHMC (2-ethylhexyl-4-methosycinnamate)	C18H26O3	Pesticides	2339	27.9	177.41
Endosulfan ether	C9H6Cl6O	Pesticides	2362	28.2	150.27
Endosulfan sulfate	C9H6Cl6O4S	Pesticides	2329	27.7	164.64
Endrin	C12H8Cl6O	Pesticides	2165	24.9	160.32
EPN	C14H14NO4PS	Pesticides	2460	29.8	173.58
Epoxiconazole	C17H13ClFN3O	Pesticides	2424	29.2	175.37
Ethalfluralin	C13H14F3N3O4	Pesticides	1648	14.8	172.65
Ethion	C9H22O4P2S4	Pesticides	2281	26.9	179.28
Ethoxyquin	C14H19NO	Pesticides	1718	16.3	155.01
Etofenprox	C25H28O3	Pesticides	2870	35.7	199.86
Famphur	C10H16NO5PS2	Pesticides	2334	27.8	168.85
Fenamiphos	C13H22NO3PS	Pesticides	2154	24.7	170.33
Fenarimol	C17H12Cl2N2O	Pesticides	2602	31.9	170.96
Fenhexamid	C14H17Cl2NO2	Pesticides	2351	28.0	164.17
Fenitrothion	C9H12NO5PS	Pesticides	1941	21.2	158.08
Fenoxycarb	C17H19NO4	Pesticides	2459	29.8	178.73
Fenpropimorph	C20H33NO	Pesticides	2178*	25.1*	184.04
Fenthion	C10H15O3PS2	Pesticides	1981	22.2	159.29
Fenvalerate Isomer I	C25H22ClNO3	Pesticides	2827	35.4	202.92

Table S2. Suspect list for GC-APCI-IMS-HRMS including the compound name, molecular formula, the family to which it belongs, experimental RI for semi-standard non-polar columns (NIST1), retention time calculated from RI NIST values, and CCS predicted by MARS.

Compound	Formula	Family	LRI NIST	RT_{calc} (min)	CCS_{pred} (Å^2)
Fenvalerate Isomer II	C25H22ClNO3	Pesticides	2827	35.1	202.92
Fipronil	C12H4Cl2F6N4OS	Pesticides	2052	23.3	181.84
Flucythrinate	C26H23F2NO4	Pesticides	2844	35.4	206.3
Fluquinconazole	C16H8Cl2FN5O	Pesticides	2729	33.8	176.04
Folpet	C9H4Cl3NO2S	Pesticides	2086	23.6	145.62
Heptachlor	C10H5Cl	Pesticides	1922	20.6	156.47
Heptachlor epoxide-a	C10H5Cl7O	Pesticides	2080	23.6	157.33
Heptachlor epoxide-b	C10H5Cl7O	Pesticides	2080	23.6	157.33
Hexachlorobenzene	C6Cl6	Pesticides	1717	16.3	137.42
Hexachlorobutadiene	C4Cl6	Pesticides	1259	6.2	134.84
Indoxocarb	C22H17ClF3N3O7	Pesticides	3044	38.0	134.84
Ioxynil octanoate	C15H17I2NO2	Pesticides	2737	33.9	180.19
Iprodione	C13H13Cl2N3O3	Pesticides	2452	29.7	170.62
Iprovalicarb	C18H28N2O3	Pesticides	2410*	29.0*	182.47
Isodrin	C12H8Cl6O	Pesticides	2015	23.0	160.34
Isopyrazam	C20H23F2N3O	Pesticides	2663	32.8	185.95
lambda-Cyhalothrin (isomer I)	C23H19ClF3NO3	Pesticides	2519	30.7	201.31
lambda-Cyhalothrin (isomer II)	C23H19ClF3NO3	Pesticides	2538	31.0	201.31
Leptophos	C13H10BrCl2O2PS	Pesticides	2495	30.3	171.77
Malathion	C10H19O6PS2	Pesticides	1962	21.8	168.04
MEPANIPYRIM	C14H13N3	Pesticides	2134	24.4	151.9
Mephosfolan	C8H16NO3PS2	Pesticides	2048*	23.3*	152.71
Metalaxyl	C15H21NO4	Pesticides	1915	20.4	161.97
Metconazole	C17H22ClN3O	Pesticides	2492*	30.3*	176.75
Methidathion	C6H11N2O4PS3	Pesticides	2100	23.8	156
Methiocarb	C11H15NO2S	Pesticides	1956	21.6	152.17
Methoxychlor	C16H15Cl3O2	Pesticides	2454*	29.7*	145.12
Metolachlor	C15H22ClNO2	Pesticides	1968	22.0	160.93
Metrafenone	C19H21BrO5	Pesticides	2643	32.5	184.2
Metribuzin	C8H14N4OS	Pesticides	1888	19.8	145.4
Mirex	C10Cl12	Pesticides	2472	30.0	
Molinate	C9H17NOS	Pesticides	1547	12.5	141.07
Octocrylene	C24H27NO2	Pesticides	2656	32.7	198.13
Omethoate	C5H12NO4PS	Pesticides	1596	13.7	137.89
Oxadixyl	C14H18N2O4	Pesticides	2280	26.9	159.48
Oxyfluorfen	C15H11ClF3NO4	Pesticides	2198	25.5	177.92
Parathion ethyl	C10H14NO5PS	Pesticides	2000	22.3	163.85
Parathion methyl	C8H10NO5PS	Pesticides	1903	20.1	153.54
Pendimethalin	C13H19N3O4	Pesticides	2046	23.3	162.92

Table S2. Suspect list for GC-APCI-IMS-HRMS including the compound name, molecular formula, the family to which it belongs, experimental RI for semi-standard non-polar columns (NIST1), retention time calculated from RI NIST values, and CCS predicted by MARS.

Compound	Formula	Family	LRI NIST	RT_{calc} (min)	CCS_{pred} ($Å^2$)
Pentachlorobenzene	C6HCl5	Pesticides	1509	11.7	133.76
Permethrine (isomero I)	C21H19ClO3	Pesticides	2711	33.5	193.56
Permethrine (isomero II)	C21H19ClO3	Pesticides	2711	33.5	193.56
Phorate	C7H17O2PS3	Pesticides	1703	16.0	151.7
Phosmet	C11H12NO4PS2	Pesticides	2452	29.7	164.1
Phosphamidon	C10H19ClNO5P	Pesticides	1811	18.2	160.79
Pirimicarb	C11H18N4O2	Pesticides	1837	18.7	154.37
Pirimiphos methyl	C11H20N3O3PS	Pesticides	1940	21.2	167.72
Procymidone	C13H11Cl2NO2	Pesticides	2088	23.6	158.04
Profenofos	C11H15BrClO3PS	Pesticides	2184	25.2	166.73
Propachlor	C11H14ClNO	Pesticides	1612	14.0	144.07
Propazine	C9H16ClN5	Pesticides	1759	17.1	153.01
Propetamphos	C10H20NO4PS	Pesticides	1777	17.5	162.2
Propham	C10H13NO2	Pesticides	1423	9.8	124.84
Propiconazole	C15H17Cl2N3O2	Pesticides	2312	27.4	174.95
Propoxur	C11H15NO3	Pesticides	1610	14.0	131.59
Propyzamide	C12H11Cl2NO	Pesticides	1786	17.7	154.1
Prosulfocarb	C14H21NOS	Pesticides	1909	20.3	163.06
Pyriproxyfen	C20H19NO3	Pesticides	2574	31.5	184.42
Quinalphos	C12H15N2O3PS	Pesticides	2086	23.6	163.21
Quintozene	C6Cl5NO2	Pesticides	1759	17.1	141.25
Resmethrin	C22H26O3	Pesticides	2399	28.8	191.45
Simazine	C7H12ClN5	Pesticides	1744	16.8	143.77
SPIROMESIFEN	C23H30O4	Pesticides	2873*	35.8*	194.48
Sulprofos	C12H19O2PS3	Pesticides	2260	26.5	173.05
Tebuconazole	C16H22ClN3O	Pesticides	2397	28.8	173.57
TEHP H (Tris(2-ethylhexyl) phosphate)	C24H51O4P	Pesticides	2463	29.8	212.05
Terbacil	C9H13ClN2O2	Pesticides	1816	18.3	126.12
Terbufos	C9H21O2PS3	Pesticides	1783	17.6	142.24
Terbumeton	C10H19N5O	Pesticides	1749	16.9	155.22
Terbumeton-desethyl	C8H15N5O	Pesticides	1726	16.5	144.75
Terbuthylazine	C9H16ClN5	Pesticides	1785	17.7	152.39
Terbuthylazine (des-ethyl)	C7H12ClN5	Pesticides	1656	15.0	142.18
Terbutryn	C10H19N5S	Pesticides	1945	21.3	158.32
Tetradifon	C12H6Cl4O2S	Pesticides	2536	31.0	163.38
Thiabendazole	C10H7N3S	Pesticides	2091	23.7	141.22
Tolclofos-methyl	C9H11Cl2O3PS	Pesticides	1899	20.0	152.19
Tolylfluanid	C8H6Cl2FNS	Pesticides	2016	23.0	165.63
trans-chlordane	C10H5Cl7	Pesticides	2089	23.7	156.14

Table S2. Suspect list for GC-APCI-IMS-HRMS including the compound name, molecular formula, the family to which it belongs, experimental RI for semi-standard non-polar columns (NIST1), retention time calculated from RI NIST values, and CCS predicted by MARS.

Compound	Formula	Family	LRI NIST	RT_{calc} (min)	CCS_{pred} (Å^2)
Triadimefon	C14H16ClN3O2	Pesticides	1999	22.9	166.29
Triflumizole	C15H15ClF3N3O	Pesticides	2057	23.4	173.82
Trifluralin	C13H16F3N3O4	Pesticides	1663	15.1	
Vinclozolin	C12H9Cl2NO3	Pesticides	1890	19.8	157.2
α-Endosulfan	C9H6Cl6O3S	Pesticides	2152	24.7	162.37
α-HCH	C6H6Cl6	Pesticides	1714	16.2	125.82
β-Endosulfan	C9H6Cl6O3S	Pesticides	2183	25.2	162.37
β-HCH	C6H6Cl6	Pesticides	1761	17.2	125.82
γ-HCH	C6H6Cl6	Pesticides	1779	18.1	125.82
δ-HCH	C6H6Cl6	Pesticides	1833	18.7	125.82

[1] National Institute of Standards and Technology (2023). NIST/EPA/NIH EI-MS Library. https://chemdata.nist.gov/dokuwiki/doku.php?id=chemdata:start. Accessed March 7, 2024.

* Estimated non-polar retention index (experimental value not available).

Table S3. Target assessment of fortified samples in spiked feed. For GC-APCI-VION-QTOF MS, positive assignments (▪), CCS > 2% (CCS), no fragmentation matched with the databaase (❶), and mass accuracy > 5 ppm (☒) . For GC-EI-QOrbitrap MS, positive assignments (▫), and no confirming ion detected (①)

Compound	Formula	Family	Fish feed 1	Fish feed 2	Fish feed 3	Ing. 1	Ing. 2	Ing. 3	Ing. 4	Ing. 5	Ing. 6
DEET	C12H17NO	Insect repellent	■ □	■ □	■ □	■ □	■ □	■ □	■ □	■ □	■ □
Cashmeran	C14H22O	Musk	■ □	■ □	■ □	■ □	■ □	■ □	■ □	■ □	■ □
Galaxolide	C17H22O	Musk	■ □	■ □	■ □	■ □	■ □	■ □	■ □	■ □	■ □
Muskone	C16H30O	Musk	■ □	■ □	■ □	■ □	■ ①	■ □	■ □	■ □	■ ①
Tonalid	C18H26O	Musk	■ □	■ □	■ □	■ □	■ □	■ □	■ □	■ □	■ □
Traseolide	C18H26O	Musk	■ □	■ □	■ □	■ □	■ □	■ □	■ □	■ □	■ □
TBEP H (Tri(2-butoxyethyl) phosphate)	C18H39O7P	OPEs	■ □	■ □	■ □	■ □	■ □	■ □	■ □	■ □	■ □
TBP H (Tributyl phosphate)	C12H27O4P	OPEs	❶ □	❶ □	■ □	■ □	■ □	❶ □	■ □	■ □	❶ □
TCEP H (Tri(2-chloroethyl) phosphate)	C6H12Cl3O4P	OPEs	■ □	■ □	■ □	■ □	■ □	■ □	■ □	■ □	■ □
TCrP H (isomer I) (Triresyl phosphate)	C21H21O4P	OPEs	□	□	□	□	□	□	□	□	①
TCrP H (isomer II) (Tricresyl phosphate)	C21H21O4P	OPEs	■ □	■ □	■ □	■ □	■ □	■ □	■ □	■ □	■ □
TCrP H (isomer III) (Tricresyl phosthate)	C21H21O4P	OPEs	■ ①	■ ①	■ ①	■ □	■ ①	■ □	■ ①	■ ①	■ ①
TDCP H (Tris(1,3- dichloro-2-propyl) phosphate)	C9H15Cl6O4P	OPEs	CCS □	CCS □	CCS □	CCS □	CCS □	CCS □	CCS □	CCS □	CCS □
TPhP H (Triphenyl phosphate)	C18H15O4P	OPEs	■ □	■ □	■ □	■ □	■ □	■ □	■ □	■ □	■ □
TPrP H (Tripropyl phosphate)	C9H21O4P	OPEs	CCS □	■ □	■ □	■ □	CCS □	■ □	□	■ □	■ □
5-Methylchrysene	C19H14	PAHs	■ □	■ □	■ □	■ □	■ □	■ □	■ □	■ □	■ □

Table S3. Target assessment of fortified samples in spiked feed. For GC-APCI-VION-QTOF MS, positive assignments (▪), CCS > 2% (CCS), no fragmentation matched with the databaase (❶), and mass accuracy > 5 ppm (☒) . For GC-EI-QOrbitrap MS, positive assignments (▫), and no confirming ion detected (①)

Compound	Formula	Family	Fish feed 1	Fish feed 2	Fish feed 3	Ing. 1	Ing. 2	Ing. 3	Ing. 4	Ing. 5	Ing. 6
Acenaphthene	C12H10	PAHs	■ □	■ □	■ □	■ □	■ □	■ □	□	❶ □	❶ □
Acenaphthylene	C12H8	PAHs	■ □	■ □	■ □	■ □	■ □	■ □	■ □	□	■ □
Anthracene	C14H10	PAHs	■ □	■ □	■ □	■ □	■ □	■ □	■ □	■ □	■ □
Benz[a]anthracene	C18H12	PAHs	■ □	■ □	■ □	■ □	■ □	■ □	■ □	■ □	■ □
benzo(c)fluorene	C17H12	PAHs	■ □	☒ □	■ □	■ □	■ □	■ □	■ □	■ □	■ □
Benzo[a]pyrene	C20H12	PAHs	■ □	■ □	■ □	■ □	■ □	■ □	■ □	■ □	❶ □
Benzo[b]fluoranthene	C20H12	PAHs	■ □	■ □	■ □	■ □	■ □	■ □	■ □	■ □	■ □
Benzo[ghi]perylene	C22H12	PAHs	■ □	■ □	■ □	■ □	CCS □	■ □	■ □	■ □	■ □
Benzo[j]fluoranthene	C20H12	PAHs	■ □	■ □	■ □	■ □	■ □	■ □	■ □	■ □	■ □
Chrysene	C18H12	PAHs	■ □	■ □	■ □	■ □	■ □	■ □	■ □	■ □	■ □
Dibenz[a,h]anthracene	C22H14	PAHs	❶ □	❶ □	❶ □	❶ □	❶ □	❶ □	❶ □	❶ □	■ □
Fluoranthene	C16H10	PAHs	■ □	■ □	■ □	■ □	■ □	■ □	■ □	■ □	■ □
Fluorene	C13H10	PAHs	■ □	■ □	■ □	■ □	■ □	■ □	■ □	■ □	■ □
Indeno[1,2,3-cd]pyrene	C22H12	PAHs	❶ □	❶ □	■	■ □	❶ □	■ □	❶ □	❶ □	❶ □
Phenanthrene	C14H10	PAHs	■ □	■ □	■ □	■ □	■ □	■ □	■ □	■ □	■ □
Pyrene	C16H10	PAHs	■ □	■ □	■ □	■ □	■ □	■ □	■ □	■ □	■ □

Table S3. Target assessment of fortified samples in spiked feed. For GC-APCI-VION-QTOF MS, positive assignments (▪), CCS > 2% (CCS), no fragmentation matched with the databaase (❶), and mass accuracy > 5 ppm (☒) . For GC-EI-QOrbitrap MS, positive assignments (▫), and no confirming ion detected (①)

Compound	Formula	Family	Fish feed 1	Fish feed 2	Fish feed 3	Ing. 1	Ing. 2	Ing. 3	Ing. 4	Ing. 5	Ing. 6
PCB 101	C12H5Cl5	PCBs	■ □	■ □	■ □	■ □	■ □	■ □	■ □	■ □	■ □
PCB 118	C12H5Cl5	PCBs	■ □	■ □	■ □	■ □	■ □	■ □	■ □	■ □	■ □
PCB 180	C12H3Cl7	PCBs	■ □	■ □	■ □	■ □	■ □	■ □	■ □	■ □	■
PCB 28	C12H7Cl3	PCBs	■ □	■ □	■ □	■ □	■ □	■ □	■ □	■ □	■ □
PCB-138	C12H4Cl6	PCBs	■ □	■ □	■ □	■ □	■ □	■ □	■ □	■ □	■ □
PCB-153	C12H4Cl6	PCBs	■ □	■ □	■ □	■ □	■ □	■ □	■ □	■ □	■ □
PCB-52	C12H6Cl4O2S	PCBs	■ □	■ □	■ □	■ □	■ □	■ □	■ □	■ □	■ □
2-Phenylphenol	C12H10O	Pesticides	■ □	■ □	■ □	■ □	■ □	■ □	■ □	■ □	■ □
4,4'-Dichlorobenzophenone	C13H8Cl2O	Pesticides	■ □	■ □	■ □	■ □	■ □	■ □	■ □	■ □	■ □
Alachlor	C14H20ClNO2	Pesticides	■ □	■ □	■ □	■ □	■ □	■ □	■ □	■ □	■ □
Aldrin	C12H8Cl6O	Pesticides	❶	❶	❶	■ □	■	❶ □	■	■ □	☒
Atrazine	C8H14ClN5	Pesticides	■ □	■ □	■ □	■ □	■ □	■ □	■ □	■ □	■ □
Atrazine deisopropyl	C5H8ClN5	Pesticides	■ □	■ □	■ □	■ □	■ □	■ □	■ □	■ □	■ □
Atrazine desethyl	C6H10ClN5	Pesticides	■ □	■ □	■ □	■ □	■ □	■ □	■ □	■ □	■ □
Azaconazole	C12H11Cl2N3O2	Pesticides	■ □	■ □	■ □	■ □	■ □	■ □	■ □	■ □	■ □
Azinphos-ethyl	C12H16N3O3PS2	Pesticides	□	□	□	□	□	□	□	□	□

Table S3. Target assessment of fortified samples in spiked feed. For GC-APCI-VION-QTOF MS, positive assignments (▪), CCS > 2% (CCS), no fragmentation matched with the databaase (❶), and mass accuracy > 5 ppm (☒) . For GC-EI-QOrbitrap MS, positive assignments (▫), and no confirming ion detected (①)

Compound	Formula	Family	Fish feed 1	Fish feed 2	Fish feed 3	Ing. 1	Ing. 2	Ing. 3	Ing. 4	Ing. 5	Ing. 6
Azinphos-methyl	C10PN3H12S2O3	Pesticides	■ □	CCS □	■ □	❶ □	❶ □	❶ □	CCS □	CCS □	❶ □
Azoxystrobin	C22H17N3O5	Pesticides	❶ □	■ □	■ □	■ □	■ □	■ □	■ □	■ □	■ □
Bifenthrin	C23H22ClF3O2	Pesticides	❶ □	❶ □	❶ □	❶ □	❶ □	❶ □	❶ □	❶ □	❶ □
Bixafen	C18H12Cl2F3N3O	Pesticides	■ □	■ □	■ □	■ □	■ □	■ □	■ □	■ □	■ □
Bromophos	C8H8BrCl2O3PS	Pesticides	■ □	■ □	■ □	■ □	■ □	■ □	■ □	■ □	■ □
Bromophos-ethyl	C10H12BrCl2O3PS	Pesticides	■ □	■ □	■ □	■ □	■ S	■ □	■ □	■ □	■ □
Bromopropylate	C17H16Br2O3	Pesticides	■ □	■ □	■ □	■ □	■ □	■ □	■ □	■ □	■ □
Bromuconazole I	C13H12BrCl2N3O	Pesticides	■ □	■ □	■ □	■ □	■ □	■ □	■ □	■ □	■ □
Bromuconazole II	C13H12BrCl2N3O	Pesticides	■ □	■ □	■ □	■ □	■ □	■ □	■ □	■ □	■ □
Buprofezin	C16H23N3OS	Pesticides	❶ □	❶ □	❶ □	■ □	❶ □	■ □	□	■ □	□
Cadusafos	C10H23O2PS2	Pesticides	■ □	■ □	■ □	■ □	■ □	■ □	■ □	■ □	■ □
Captan	C9H7Cl2NO2S	Pesticides		❶	❶ ①	■	❶	❶ □	❶	❶	❶
Carbofuran	C12H15NO3	Pesticides	①	①	①	□	①	□	①	□	□
Carfentrazone ethyl	C15H14Cl2F3N3O3	Pesticides	■ □	■ □	■ □	■ □	■ □	■ □	■ □	■ □	■ □
Chinomethionate	C10H6N2OS2	Pesticides	❶	■ □	■	■ □	■	■ □	■ □	■ □	■ □
Chlorfenapyr	C15H11BrClF3N2O	Pesticides	■ □	■ □	■ □	■ □	■ □	■ □	■ □	■ □	■ □

Table S3. Target assessment of fortified samples in spiked feed. For GC-APCI-VION-QTOF MS, positive assignments (▪), CCS > 2% (CCS), no fragmentation matched with the databaase (❶), and mass accuracy > 5 ppm (☒) . For GC-EI-QOrbitrap MS, positive assignments (▫), and no confirming ion detected (①)

Compound	Formula	Family	Fish feed 1	Fish feed 2	Fish feed 3	Ing. 1	Ing. 2	Ing. 3	Ing. 4	Ing. 5	Ing. 6
Chlorfenson	C12H8Cl2O3S	Pesticides	■ □	■ □	■ □	■ □	■ □	■ □	■ □	■ □	■ □
Chloropyriphos-methyl	C7H7Cl3NO3PS	Pesticides	■ □	■ □	■ □	■ □	■ □	■ □	■ □	■ □	■ □
Chlorothalonil	C8Cl4N2	Pesticides	■ □	■ □	□	■ □		■ □	■ □	■ □	■ □
Chlorpropham	C10H12ClNO2	Pesticides	□	□	□	□	□	□	□	□	□
Chlorpyrifos-ethyl	C9H11Cl3NO3PS	Pesticides	■ □	■ □	■ □	■ □	■ □	■ □	■ □	■ □	■ □
Clodinafop-propargyl	C17H13ClFNO4	Pesticides	■ □	■ □	■ □	■ □	■ □	■ □	■ □	■ □	■ □
Clofenvinfos (isomer I)	C12H14Cl3O4P	Pesticides	■ □	■ □	■ □	■ □	■ □	■ □	■ □	■ □	■ □
Coumaphos	C14H16ClO5PS	Pesticides	■ □	■ □	■ □	■ □	■ □	■ □	■ □	■ □	■ □
Cyanazine	C9H13ClN6	Pesticides	■ □	■ □	■ □	■ □	■	■ □	■ □	■ □	■ □
Cyanophos	C9H10NO3PS	Pesticides	■ □	■ □	■ □	■ □	■ □	■ □	■ □	■ □	■ □
Cyproconazole	C15H18ClN3O	Pesticides	■ □	■ □	■ □	■ □	■ □	■ □	■ □	■ □	■ □
Cyprodinil	C14H15N3	Pesticides	■ □	■ □	■ □	■ □	■ □	■ □	■ □	■ □	■ □
DDD	C14H10Cl4	Pesticides	■ □	■ □	■ □	■ □	■ □	■ □	■ □	■ □	■ □
DDE	C14H8Cl4	Pesticides	■ □	■ □	■ □	■ □	■ □	■ □	■ □	■ □	■ □
DDT	C14H9Cl5	Pesticides	■ □	■ □	■ □	■ □	■ □	■ □	■ □	■ □	■ □
Deltamethrin	C22H19Br2NO3	Pesticides		□	□	❶ □	□	❶ □	❶ □	❶ □	❶

Table S3. Target assessment of fortified samples in spiked feed. For GC-APCI-VION-QTOF MS, positive assignments (▪), CCS > 2% (CCS), no fragmentation matched with the databaase (❶), and mass accuracy > 5 ppm (☒) . For GC-EI-QOrbitrap MS, positive assignments (▫), and no confirming ion detected (①)

Compound	Formula	Family	Fish feed 1	Fish feed 2	Fish feed 3	Ing. 1	Ing. 2	Ing. 3	Ing. 4	Ing. 5	Ing. 6
Diazinone	C12H21N2O3PS	Pesticides	■ □	■ □	■ □	■ □	■ □	■ □	■ □	■ □	■ □
Dichlofenthion	C10H13Cl2O3PS	Pesticides	■ □	■ □	■ □	■ □	■ □	■ □	■ □	■ □	■ □
Dichloran	C6H4Cl2N2O2	Pesticides	■ □	■ □	■ □	■ □	■ □	■ □	■ □	■ □	■ □
Dichlorvos	C4H7Cl2O4P	Pesticides	■ □	■ □	■ □	■ □	■ □	■ □	■ □	■ □	■ □
Dicofol	C14H9Cl5O	Pesticides	■ □	■ □	■ □	■ □	■ □	■ □	■ □	■ □	■ □
Dicrotophos	C8H16NO5P	Pesticides	■ □	■ □	■ □	■ □	■ □	■ □	■ □	■ □	■ □
Dieldrin	C12H8Cl6O	Pesticides	■ □	■ □	■ □	■ □	■ □	■ □	■ □	■ □	■ □
Difenoconazole	C19H17Cl2N3O3	Pesticides	■ □	■ □	■ □	■ □	■ □	■ □	■ □	■ □	■ □
Diflufenican	C19H11F5N2O2	Pesticides	■ □	■ □	■ □	■ □	■ □	■ □	■ □	■ □	■ □
Dimethachlor	C13H18ClNO2	Pesticides	■ □	■ □	■ □	■ □	■ □	❶ □	■ □	❶ □	■ □
Dimethoate	C5H12NO3PS2	Pesticides	□	■ □	■ □	■ □	□	■ □	■ □	■ □	■ □
Dioxathion	C12H26O6P2S4	Pesticides	■ □	■ □	■ □	■ □	■ □	■ □	■ □	■ □	■ □
Diphenylamine	C12H11N	Pesticides	■ □	■ □	■ □	■ □	■ □	■ □	■ □	■ □	■ □
EHMC (2-ethylhexyl-4-methosycinnamate)	C18H26O3	Pesticides	□	□	□	□	□	□	□	□	□
Endosulfan ether	C9H6Cl5[37Cl]O	Pesticides	■ □	■ □	■ □	■ □	■ □	■ □	■ □	■ □	■ □
Endosulfan sulfate	C9H6Cl6O4S	Pesticides	□	□	□	□	□	□	□	□	□

Table S3. Target assessment of fortified samples in spiked feed. For GC-APCI-VION-QTOF MS, positive assignments (▪), CCS > 2% (CCS), no fragmentation matched with the databaase (❶), and mass accuracy > 5 ppm (☒) . For GC-EI-QOrbitrap MS, positive assignments (▫), and no confirming ion detected (①)

Compound	Formula	Family	Fish feed 1	Fish feed 2	Fish feed 3	Ing. 1	Ing. 2	Ing. 3	Ing. 4	Ing. 5	Ing. 6
Endrin	C12H8Cl6O	Pesticides	❶ □	■ □	❶ □	■ □	■ □	■ □	❶ □	■ □	❶ □
EPN	C14H14NO4PS	Pesticides	■ □	■ □	■ □	■ □	■ □	■ □	■ □	■ □	■ □
Epoxiconazole	C17H13ClFN3O	Pesticides	■ □	■ □	■ □	■ □	■ □	■ □	■ □	■ □	■ □
Ethalfluralin	C13H14F3N3O4	Pesticides	■ □	■ □	■ □	■ □	■ □	■ □	■ □	■ □	■ □
Ethion	C9H22O4P2S4	Pesticides	■ □	■ □	■ □	■ □	■ □	■ □	■ □	■ □	■ □
Ethoxyquin	C14H19NO	Pesticides	■ □	■ □	■ □	■ □	■ □	■ □	■ □	■ □	■ □
Etofenprox	C25H28O3	Pesticides	□	□	□	□	□	□	□	□	□
Famphur	C10H16NO5PS2	Pesticides	■ □	■ □	■ □	■ □	■ □	■ □	■ □	■ □	■ □
Fenamiphos	C13H22NO3PS	Pesticides		□	□	■ □	■ □	□	■	■ □	□
Fenarimol	C17H12Cl2N2O	Pesticides	■ □	■ □	■ □	■ □	■ □	■ □	■ □	■ □	■ □
Fenhexamid	C14H17Cl2NO2	Pesticides	■ ①	■ ①	■ ①	■ ①	■ ①	■ □	■ ①	■ ①	■ ①
Fenitrothion	C9H12NO5PS	Pesticides	■ □	■ □	■ □	■ □	■ □	■ □	■ □	■ □	■ □
Fenoxycarb	C17H19NO4	Pesticides	■ □	■ □	■ □	■ □	■ □	■ □	■ □	■ □	❶ □
Fenpropimorph	C20H33NO	Pesticides	■ □	■ □	■ □	■ □	■ □	■ □	■ □	■ □	■ □
Fenthion	C10H15O3PS2	Pesticides	■ □	■ □	■ □	■ □	■ □	■ □	■ □	■ □	■ □
Fenvalerate Isomer I	C25H22ClNO3	Pesticides	❶ □	■ □	■ □	■ □	■ □	■ □	■ □	■ □	■ □

Table S3. Target assessment of fortified samples in spiked feed. For GC-APCI-VION-QTOF MS, positive assignments (▪), CCS > 2% (CCS), no fragmentation matched with the databaase (❶), and mass accuracy > 5 ppm (☒) . For GC-EI-QOrbitrap MS, positive assignments (▫), and no confirming ion detected (①)

Compound	Formula	Family	Fish feed 1	Fish feed 2	Fish feed 3	Ing. 1	Ing. 2	Ing. 3	Ing. 4	Ing. 5	Ing. 6
Fenvalerate Isomer II	C25H22ClNO3	Pesticides	❶ □	■ □	■ □	■ □	□	■ □	■ □	■ □	❶ □
Fipronil	C12H4Cl2F6N4O S	Pesticides	■ □	■ □	■ □	■ □	■ □	■ □	■ □	■ □	■ □
Flucythrinate	C26H23F2NO4	Pesticides	❶ □	❶ □	■ □	■ □	■ □	■ □	■ □	■ □	■ □
Fluquinconazole	C16H8Cl2FN5O	Pesticides	■ □	■ □	■ □	■ □	■ □	■ □	■ □	■ □	■ □
Folpet	C9H4Cl3NO2S	Pesticides	❶ □	❶ □	❶ □	❶ □	❶	❶ □	❶ □	❶ □	❶ □
Heptachlor	C10H5Cl	Pesticides	□	■ □	■ □	■ □	■ □	■ □	■ □	■ □	■ □
Heptachlor epoxide-a	C10H5Cl7O	Pesticides	□	■ □	■ □	■ □	■ □	■ □	■ □	■ □	❶ □
Heptachlor epoxide-b	C10H5Cl7O	Pesticides	■ □	■ □	■ □	■ □	■ □	■ □	■ □	■ □	■ □
Hexachlorobenzene	C6Cl6	Pesticides	■ □	■ □	■ □	■ □	■ □	■ □	■ □	■ □	■ □
Hexachlorobutadiene	C4Cl6	Pesticides			■ □	■	■ □				■ □
Indoxocarb	C22H17ClF3N3O 7	Pesticides	□	■ □	■ □	■ □	■ □	■ □	■ □	■ □	■ □
Ioxynil octanoate	C15H17I2NO2	Pesticides	■ ①	■ ①	■ ①	■ ①	■	■ ①	■ ①	■ □	■ □
Iprodione	C13H13Cl2N3O3	Pesticides	■ □	❶ □	■ □	■ □	■ □	■ □	■ □	■ □	■ □
Iprovalicarb	C18H28N2O3	Pesticides	□	□	□	□	□	□	□	□	□
Isodrin	C12H8Cl6O	Pesticides	■ □	■ □	■ □	■ □	■ □	■ □	■ □	■ □	■ □
Isopyrazam	C20H23F2N3O	Pesticides	■ □	■ □	■ □	■ □	■ □	■ □	■ □	■ □	■ □

Table S3. Target assessment of fortified samples in spiked feed. For GC-APCI-VION-QTOF MS, positive assignments (▪), CCS > 2% (CCS), no fragmentation matched with the databaase (❶), and mass accuracy > 5 ppm (☒) . For GC-EI-QOrbitrap MS, positive assignments (▫), and no confirming ion detected (①)

Compound	Formula	Family	Fish feed 1	Fish feed 2	Fish feed 3	Ing. 1	Ing. 2	Ing. 3	Ing. 4	Ing. 5	Ing. 6
lambda-Cyhalothrin (isomer I)	C23H19ClF3NO3	Pesticides	❶ □	❶ □	■ □	■ □	□	■ □	❶ □	■ □	❶ □
lambda-Cyhalothrin (isomer II)	C23H19ClF3NO3	Pesticides	❶ □	❶ □	■ □	❶ □	□	❶ □	■ □	❶ □	❶ □
Leptophos	C13H10BrCl2O2PS	Pesticides	■ □	■ □	■ □	■ □	■ □	■ □	■ □	■ □	■ □
Malathion	C10H19O6PS2	Pesticides	□	□	□	□	□	□	□	□	□
MEPANIPYRIM	C14H13N3	Pesticides	■ □	■ □	■ □	■ □	■ □	■ □	■ □	■ □	■ □
Mephosfolan	C8H16NO3PS2	Pesticides	■ □	■ □	■ □	■ □	☒ □	■ □	■ □	■ □	■ □
Metalaxyl	C15H21NO4	Pesticides	■ □	■ □	■ □	■ □	■ □	■ □	■ □	■ □	■ □
Metconazole	C17H22ClN3O	Pesticides	■ □	■ □	■ □	■ □	■ □	■ □	❶ □	■ □	■ □
Methidathion	C6H11N2O4PS3	Pesticides	□	□	□	□	□	□	□	□	□
Methiocarb	C11H15NO2S	Pesticides	□	□	□	□	□	□	□	□	□
Methoxychlor	C16H15Cl3O2	Pesticides	■ □	■ □	■ □	■ □	■ □	■ □	■ □	■ □	■ □
Metolachlor	C15H22ClNO2	Pesticides	■ □	■ □	■ □	■ □	■ □	■ □	■ □	■ □	■ □
Metrafenone	C19H21BrO5	Pesticides	■ □	■ □	■ □	■ □	■ □	■ □	■ □	■ □	■ □
Metribuzin	C8H14N4OS	Pesticides	■ □	■ □	■ □	■ □	■ □	■ □	■ □	■ □	■ □
Mirex	C10Cl12	Pesticides	■ □	■ □	■ □	■ □	■ □	■ □	■ □	■ □	□
Molinate	C9H17NOS	Pesticides	■ □	■ □	■ □	■ □	■ □	■ □	■ □	■ □	■ □

Table S3. Target assessment of fortified samples in spiked feed. For GC-APCI-VION-QTOF MS, positive assignments (▪), CCS > 2% (CCS), no fragmentation matched with the databaase (❶), and mass accuracy > 5 ppm (☒) . For GC-EI-QOrbitrap MS, positive assignments (▫), and no confirming ion detected (①)

Compound	Formula	Family	Fish feed 1	Fish feed 2	Fish feed 3	Ing. 1	Ing. 2	Ing. 3	Ing. 4	Ing. 5	Ing. 6
Naphthalene	C10H8	Pesticides		①	□	①	①				□
Octocrylene	C24H27NO2	Pesticides	□	□	□	□	□	□	□	□	□
Omethoate	C5H12NO4PS	Pesticides	■ □	■ □	■ □	■ □	■ □	■ □	■ □	■ □	■ □
Oxadixyl	C14H18N2O4	Pesticides	■ □	❶ □	■ □	■ □	■ □	■ □	❶ □	■ □	■ □
Oxyfluorfen	C15H11ClF3NO4	Pesticides	■ □	■ □	❶ □	■ □	■ □	❶ □	■ □	■ □	❶ □
Parathion ethyl	C10H14NO5PS	Pesticides	■ □	■ □	■ □	■ □	■ □	■ □	■ □	■ □	■ □
Parathion methyl	C8H10NO5PS	Pesticides	■ □	■ □	■ □	■ □	■ □	■ □	■ □	■ □	■ □
Pendimethalin	C13H19N3O4	Pesticides	□	□	□	□	□	□	□	□	□
Pentachlorobenzene	C6HCl4[37Cl]	Pesticides	■ □	■ □	■ □	■ □	■ □	■ □	■ □	■ □	■ □
Permethrine (isomero I)	C21H19ClO3	Pesticides	①	①	①	■ □	❶ ①	■ □	①	■ ①	■ ①
Permethrine (isomero II)	C21H19ClO3	Pesticides	□	❶ □	❶ □	■ ①	❶ □	❶ □	❶ □	❶ □	❶ □
Phorate	C7H17O2PS3	Pesticides	□	□	□	□	□	□	□	□	□
Phosmet	C11H12NO4PS2	Pesticides	□	□	□	■ □	□	■ □	□	■ □	☒ □
Phosphamidon	C10H19ClNO5P	Pesticides	■ □	■ □	■ □	■ □	■ □	■ □	■ □	■ □	■ □
Pirimicarb	C11H18N4O2	Pesticides	■ □	■ □	■ □	■ □	■ □	■ □	■ □	■ □	■ □
Pirimiphos methyl	C11H20N3O3PS	Pesticides	■ □	■ □	■ □	■ □	■ □	■ □	■ □	■ □	■ □

Table S3. Target assessment of fortified samples in spiked feed. For GC-APCI-VION-QTOF MS, positive assignments (▪), CCS > 2% (CCS), no fragmentation matched with the databaase (❶), and mass accuracy > 5 ppm (☒) . For GC-EI-QOrbitrap MS, positive assignments (▫), and no confirming ion detected (①)

Compound	Formula	Family	Fish feed 1	Fish feed 2	Fish feed 3	Ing. 1	Ing. 2	Ing. 3	Ing. 4	Ing. 5	Ing. 6
Procymidone	C13H11Cl2NO2	Pesticides	CCS □	CCS □	CCS □	CCS □	CCS □	CCS □	CCS □	CCS □	CCS □
Profenofos	C11H15[81Br]ClO 3PS	Pesticides	■ □	■ □	■ □	■ □	■ □	■ □	■ □	■ □	■ □
Propachlor	C11H14ClNO	Pesticides	■ □	■ □	■ □	■ □	■ □	■ □	■ □	■ □	■ □
Propazine	C9H16ClN5	Pesticides	■ □	■ □	■ □	■ □	■ □	■ □	■ □	■ □	■ □
Propetamphos	C10H20NO4PS	Pesticides	□	□	□	❶ □	□	❶ □	❶ □	❶ □	❶ □
Propham	C10H13NO2	Pesticides	□	□	□	□	□	□	□	□	□
Propiconazole	C15H17Cl2N3O2	Pesticides	■ □	■ □	❶ □	■ □	■ □	■ □	■ □	■ □	■ □
Propoxur	C11H15NO3	Pesticides	□	□	□	□	□	□	□	□	□
Propyzamide	C12H11Cl2NO	Pesticides	■ □	■ □	■ □	■ □	■ □	■ □	■ □	■ □	■ □
Prosulfocarb	C14H21NOS	Pesticides	■ □	■ □	■ □	■ □	■ □	■ □	❶ □	■ □	■ □
Pyriproxyfen	C20H19NO3	Pesticides	■ □	■ □	■ □	■ □	■ □	■ □	■ □	■ □	■ □
Quinalphos	C12H15N2O3PS	Pesticides	■ □	■ □	■ □	■ □	■ □	■ □	■ □	■ □	■ □
Quintozene	C6Cl4[37Cl]NO2	Pesticides	■ □	■ □	■ □	❶ □	■ □	■ □	■ □	■ □	■ □
Resmethrin	C22H26O3	Pesticides	□	□	□	■ □	■ □	□	❶ □	■ □	□
Simazine	C7H12ClN5	Pesticides	■ □	■ □	■ □	■ □	■ □	■ □	■ □	■ □	■ □
SPIROMESIFEN	C23H30O4	Pesticides			□	□		□		□	□

Table S3. Target assessment of fortified samples in spiked feed. For GC-APCI-VION-QTOF MS, positive assignments (▪), CCS > 2% (CCS), no fragmentation matched with the databaase (❶), and mass accuracy > 5 ppm (☒) . For GC-EI-QOrbitrap MS, positive assignments (▫), and no confirming ion detected (①)

Compound	Formula	Family	Fish feed 1	Fish feed 2	Fish feed 3	Ing. 1	Ing. 2	Ing. 3	Ing. 4	Ing. 5	Ing. 6
Sulprofos	C12H19O2PS3	Pesticides	■ □	■ □	■ □	■ □	■ □	■ □	■ □	■ □	■ □
Tebuconazole	C16H22ClN3O	Pesticides	■ □	■ □	■ □	■ □	■ □	■ □	■ □	■ □	■ □
TEHP H (Tris(2-ethylhexyl) phosphate)	C24H51O4P	Pesticides	□	□	□	□	□	□	□	□	□
Terbacil	C9H13ClN2O2	Pesticides	■ □	■ □	■ □	■ □	■ □	■ □	■ □	■ □	■ □
Terbufos	C9H21O2PS3	Pesticides	■ □	■ □	■ □	■ □	■ □	■ □	■ □	■ □	■ □
Terbumeton	C10H19N5O	Pesticides	■ □	■ □	■ □	■ □	■ □	■ □	■ □	■ □	■ □
Terbumeton-desethyl	C8H15N5O	Pesticides	■ □	■ □	■ □	■ □	■ □	■ □	■ □	■ □	■ □
Terbuthylazine	C9H16ClN5	Pesticides	■ □	■ □	■ □	■ □	■ □	■ □	■ □	■ □	■ □
Terbuthylazine (des-ethyl)	C7H12ClN5	Pesticides	■ □	■ □	■ □	■ □	■ □	■ □	■ □	■ □	■ □
Terbutryn	C10H19N5S	Pesticides	■ □	■ □	■ □	■ □	■ □	■ □	■ □	■ □	■ □
Tetradifon	C12H6Cl4O2S	Pesticides	■ □	■ □	■ □	■ □	■ □	■ □	■ □	■ □	■ □
Thiabendazole	C10H7N3S	Pesticides	■ □	■ □	■ □	■ □	■ □	■ □	■ □	■ □	■ □
Tolclofos-methyl	C9H11Cl2O3PS	Pesticides	■ □	■ □	■ □	■ □	■ □	■ □	■ □	■ □	■ □
Tolylfluanid	C8H6Cl2FNS	Pesticides	■ □	■ □	■ □	■ □	■ □	■ □	■ □	■ □	■ □
trans-Chlordane	C10H5Cl7	Pesticides	■ □	■ □	■ □	■ □	■	■ □	■ □	■ □	■ □
Triadimefon	C14H16ClN3O2	Pesticides	■ □	■ □	■ □	■ □	■ □	■ □	■ □	■ □	■ □

Table S3. Target assessment of fortified samples in spiked feed. For GC-APCI-VION-QTOF MS, positive assignments (▪), CCS > 2% (CCS), no fragmentation matched with the databaase (❶), and mass accuracy > 5 ppm (☒) . For GC-EI-QOrbitrap MS, positive assignments (▫), and no confirming ion detected (①)

Compound	Formula	Family	Fish feed 1	Fish feed 2	Fish feed 3	Ing. 1	Ing. 2	Ing. 3	Ing. 4	Ing. 5	Ing. 6
Triflumizole	C15H15ClF3N3O	Pesticides	■ □	■ □	■ □	■ □	■ □	■ □	❶ □	■ □	■ □
Trifluralin	C13H16F3N3O4	Pesticides	■ □	■ □	■ □	■ □	■ □	■ □	■ □	■ □	■ □
Vinclozolin	C12H9Cl2NO3	Pesticides	❶ □	❶ □	❶ □	❶ □	■ □	❶ □	❶ □	❶ □	❶ □
α-Endosulfan	C9H6Cl6O3S	Pesticides	■ □	■ □	■ □	■ □	□	■ □	■ □	■ □	■ □
α-HCH	C6H6Cl6	Pesticides	■ □	■ □	■ □	■ □	■ □	■ □	■ □	■ □	■ □
β-Endosulfan	C9H6Cl6O3S	Pesticides	■ □	■ □	■ □	■ □	■ □	■ □	■ □	■ □	■ □
β-HCH	C6H6Cl6	Pesticides	■ □	■ □	■ □	■ □	■ □	■ □	■ □	■ □	■ □
γ-HCH	C6H6Cl6	Pesticides	■ □	■ □	■ □	■ □	■ □	■ □	■ □	■ □	■ □
δ-HCH	C6H6Cl6	Pesticides	■ □	■ □	■ □	■ □	■ □	■ □	■ □	■ □	■ □

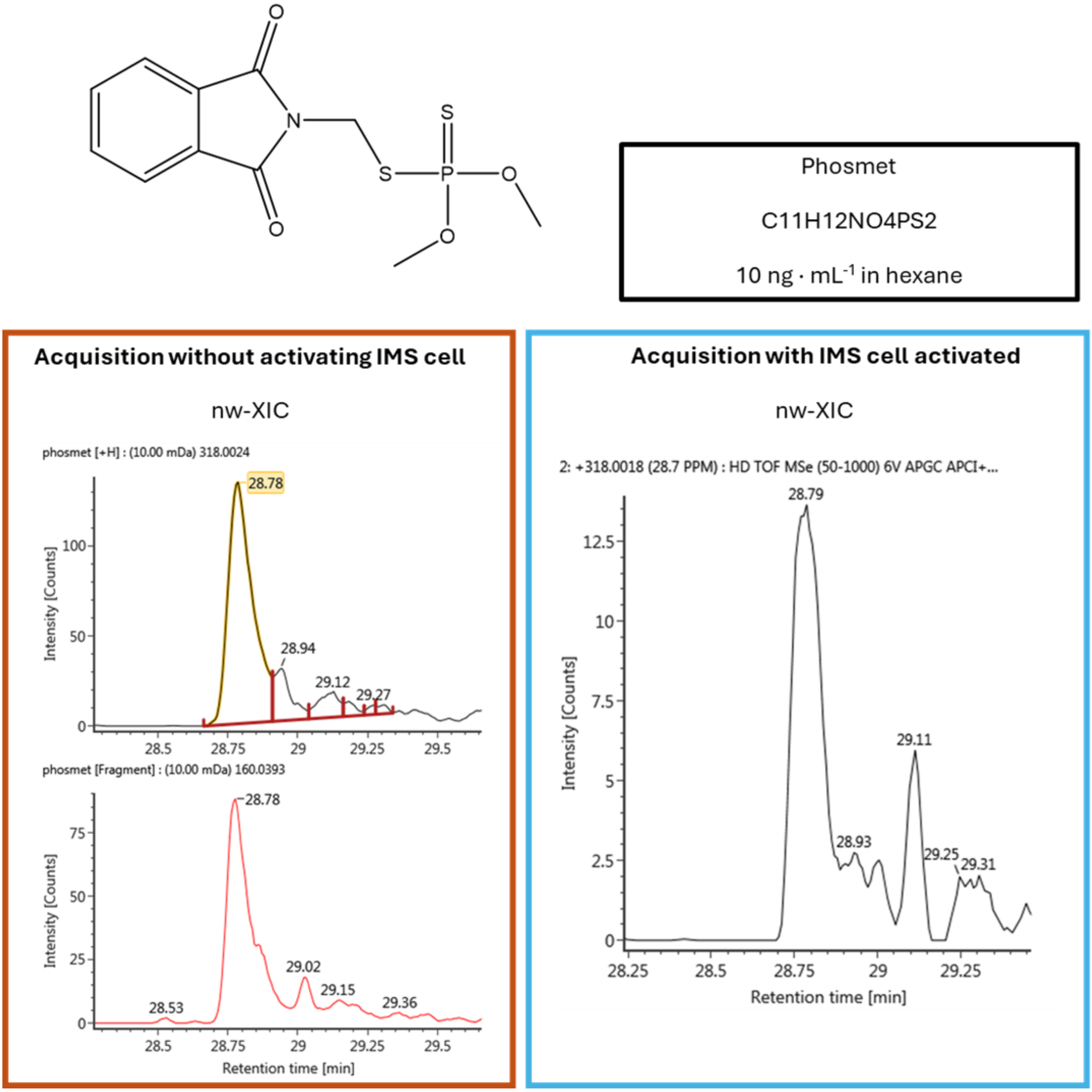

Figure S1. (Left) Narrow window-extracted ion chromatogram (nw-XIC) of phosmet at 10 ng · mL^{-1} in hexane acquired in GC-APCI-IMS-QTOF MS in MS^{E} mode. (Right) Narrow window-extracted ion chromatogram (nw-XIC) of phosmet at 10 ng • mL-1 in hexane acquired in GC-APCI-IMS-QTOF MS in HDMS^{E} mode.

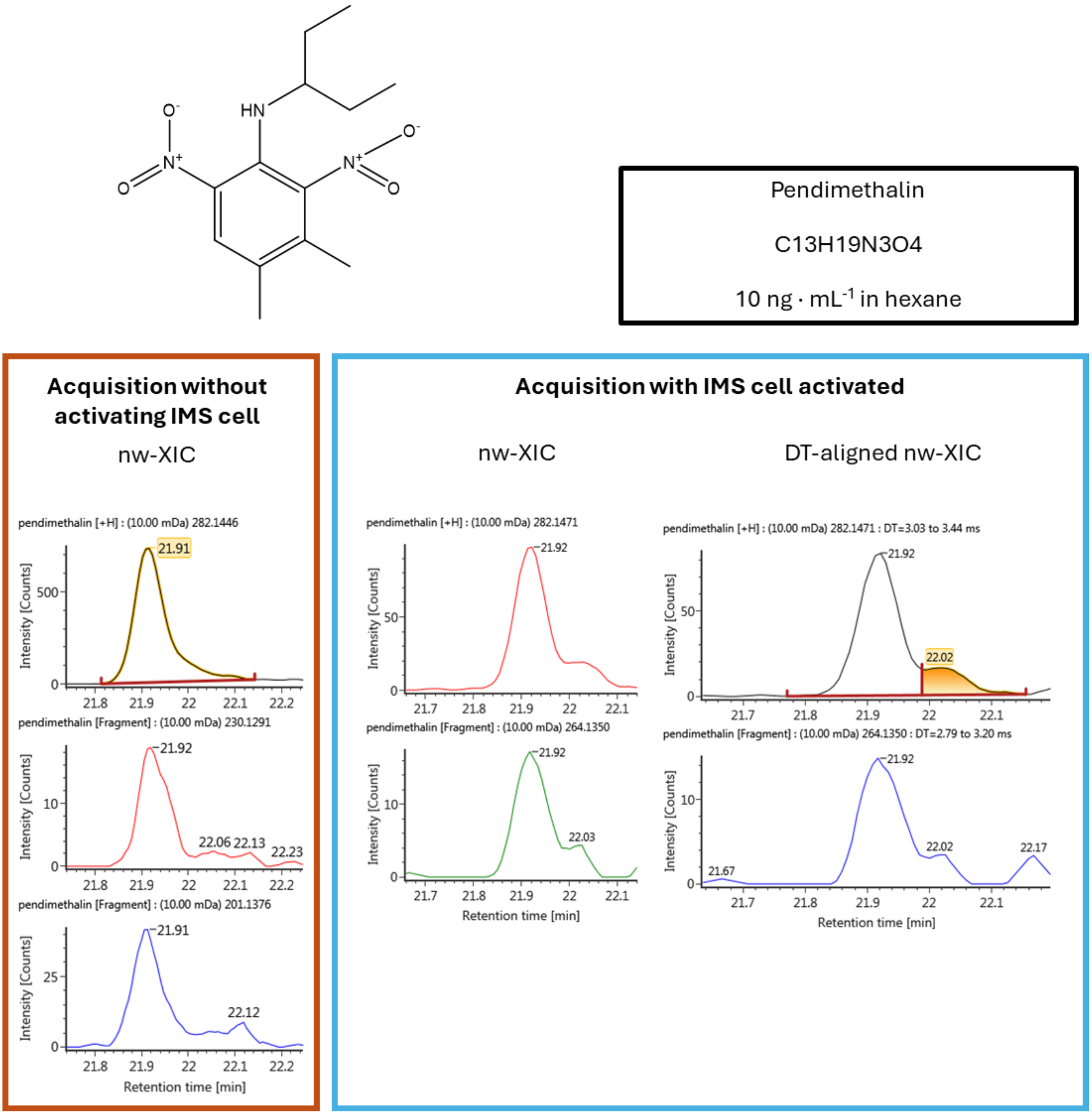

Figure S2. (Left) Narrow window-extracted ion chromatogram (nw-XIC) of pendimethalin at 10 ng · mL^{-1} in hexane acquired in GC-APCI-IMS-QTOF MS in MS^E mode. (Right) Narrow window-extracted ion chromatogram (nw-XIC) and drift time aligned nw-XIC of pendimethalin at 10 ng · mL^{-1} in hexane acquired in GC-APCI-IMS-QTOF MS in $HDMS^E$ mode.

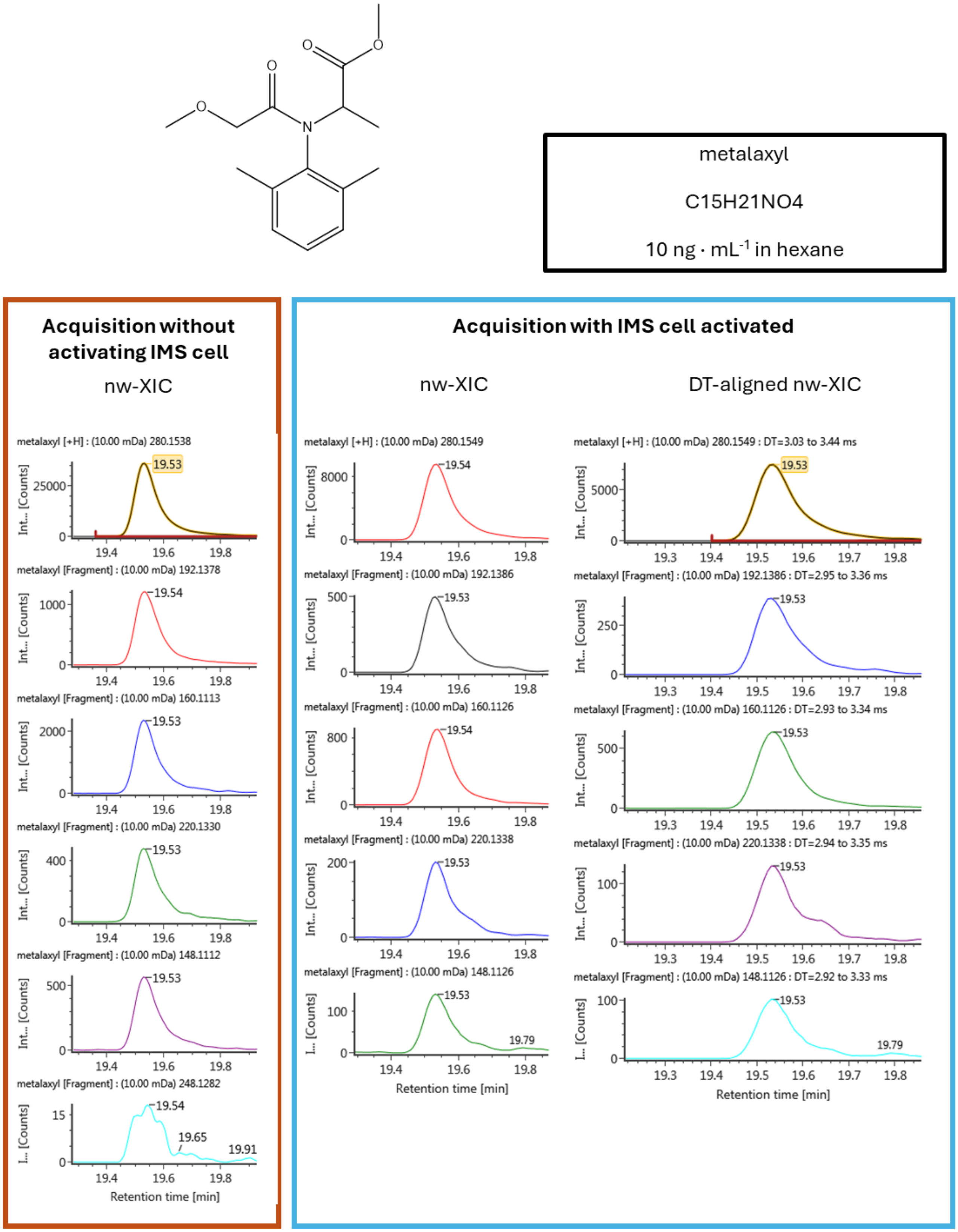

Figure S3. (Left) Narrow window-extracted ion chromatogram (nw-XIC) of metalaxyl at 10 ng · mL^{-1} in hexane acquired in GC-APCI-IMS-QTOF MS in MS^{E} mode. (Right) Narrow window-extracted ion chromatogram (nw-XIC) and drift time aligned nw-XIC of metalaxyl at 10 ng · mL^{-1} in hexane acquired in GC-APCI-IMS-QTOF MS in HDMS^{E} mode.

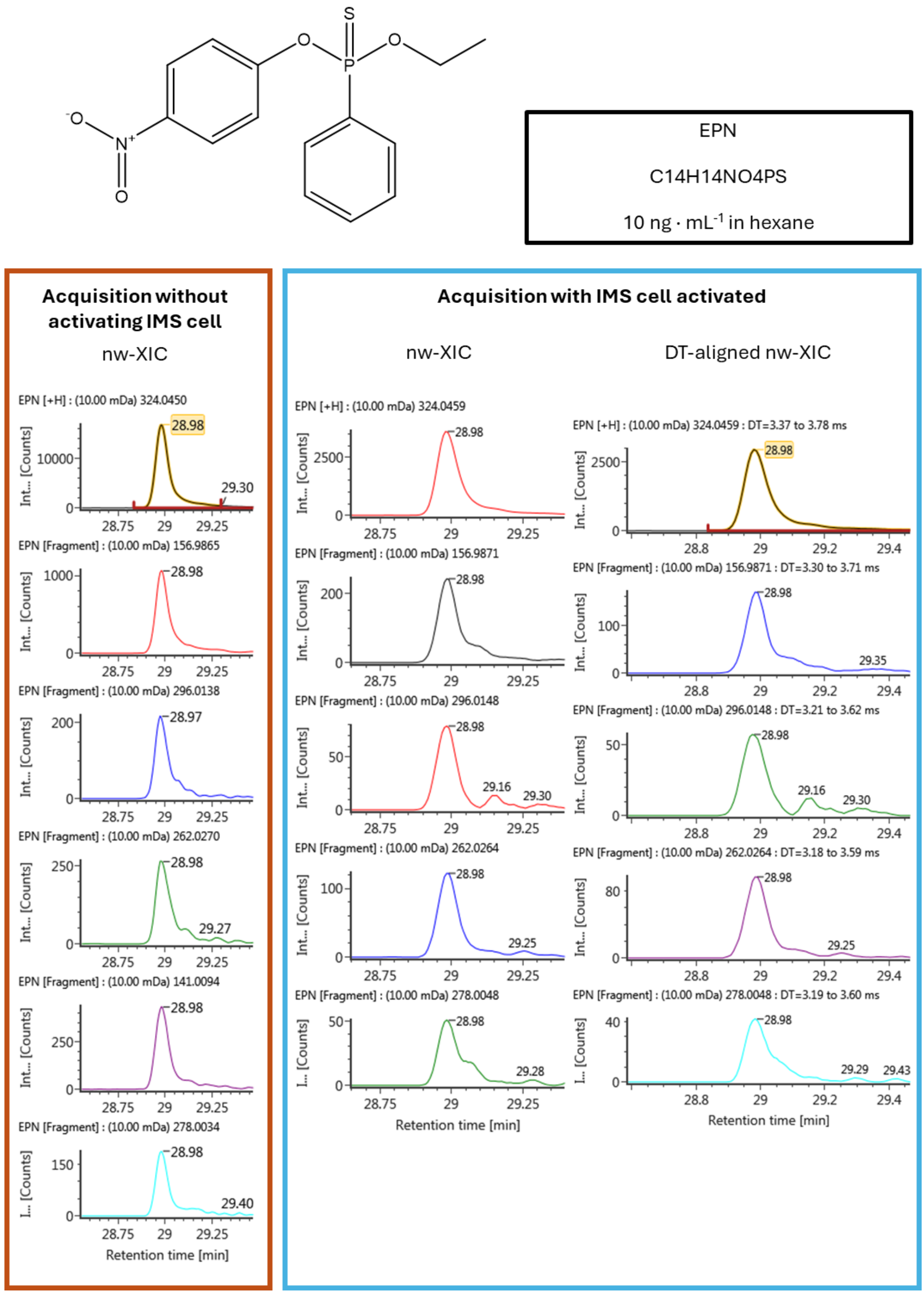

Figure S4. (Left) Narrow window-extracted ion chromatogram (nw-XIC) of EPN at 10 ng · mL^{-1} in hexane acquired in GC-APCI-IMS-QTOF MS in MS^E mode. (Right) Narrow window-extracted ion chromatogram (nw-XIC) and drift time aligned nw-XIC of EPN at 10 ng · mL^{-1} in hexane acquired in GC-APCI-IMS-QTOF MS in HDMS^E mode.

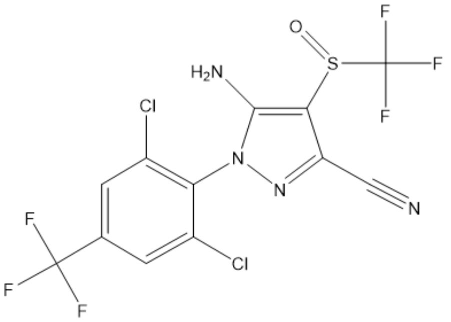

Fipronil

C12H4Cl2F6N4OS

10 ng · mL^{-1} in hexane

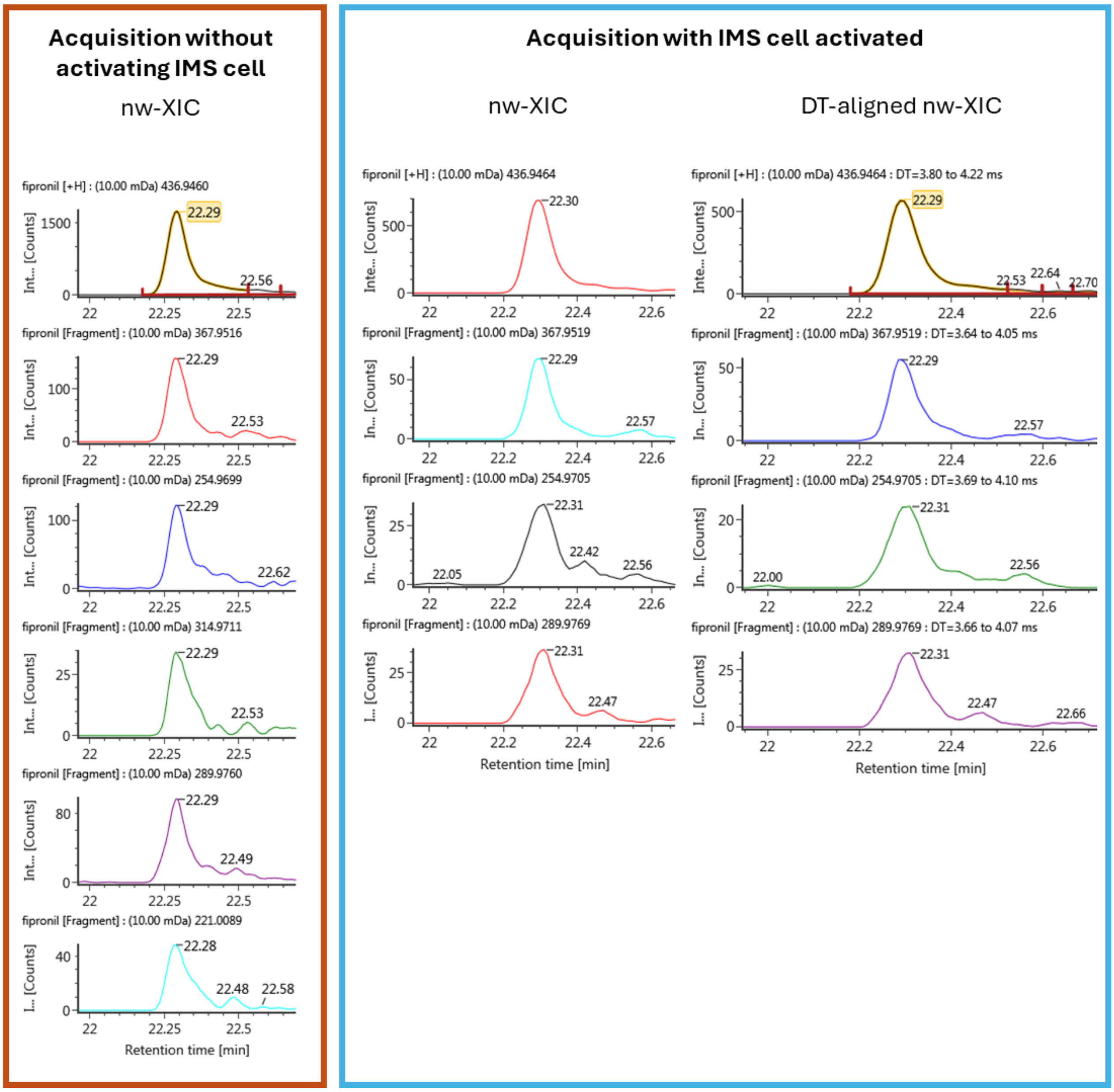

Figure S5. (Left) Narrow window-extracted ion chromatogram (nw-XIC) of firpronil at 10 ng · mL^{-1} in hexane acquired in GC-APCI-IMS-QTOF MS in MS^{E} mode. (Right) Narrow window-extracted ion chromatogram (nw-XIC) and drift time aligned nw-XIC of fipronil at 10 ng · mL^{-1} in hexane acquired in GC-APCI-IMS-QTOF MS in HDMS^{E} mode.

A)

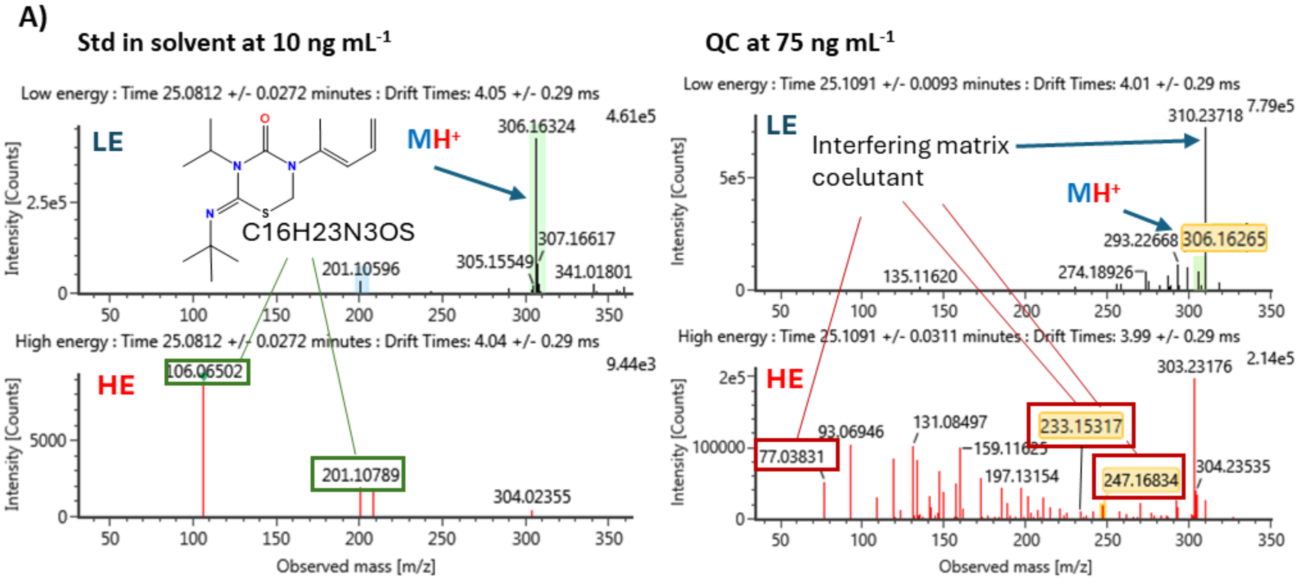

B)

Observed m/z	Mass error (mDa)	Detector co...	Structure
77.03831	-0.8	3718	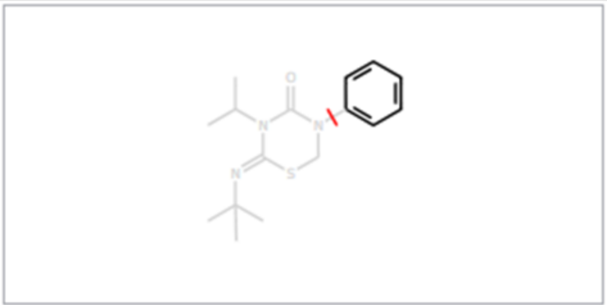
247.16834	-0.1	2357	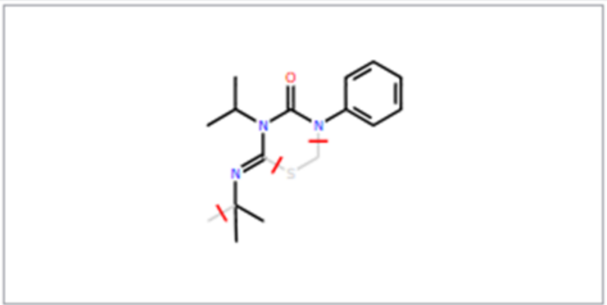
233.15317	0.4	1324	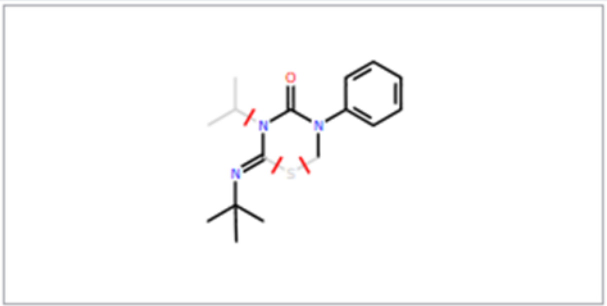

Figure S6. A) Low- and high-energy spectra (HDMS^E) of buprofezin in GC-APCI-IMS QTOF MS in solvent (left) and spiked sample, feed fish 1 (right), showing analyte fragments (green rectangle) and the false assignations of the in silico fragmentation tool (red rectangle). B) False assignations structures and mass errors.

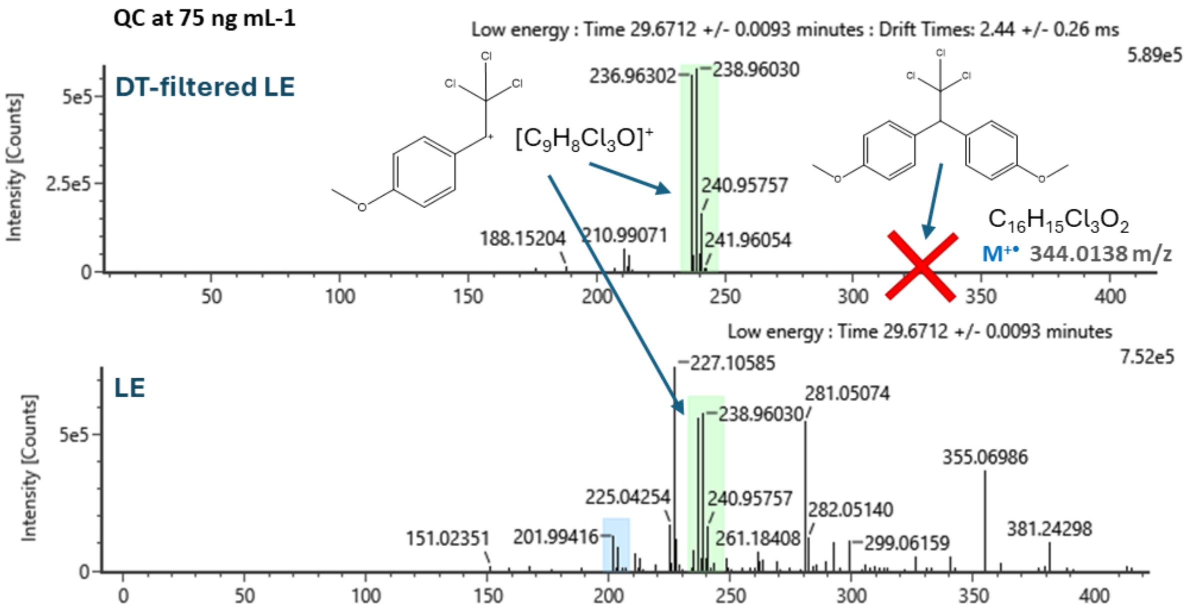

Figure S7. Drift time-filtered LE and LE spectra of methoxychlor in the spiked sample (fish feed 1), red cross indicates the absence of the molecular/(de)protonated ion of the analyte.

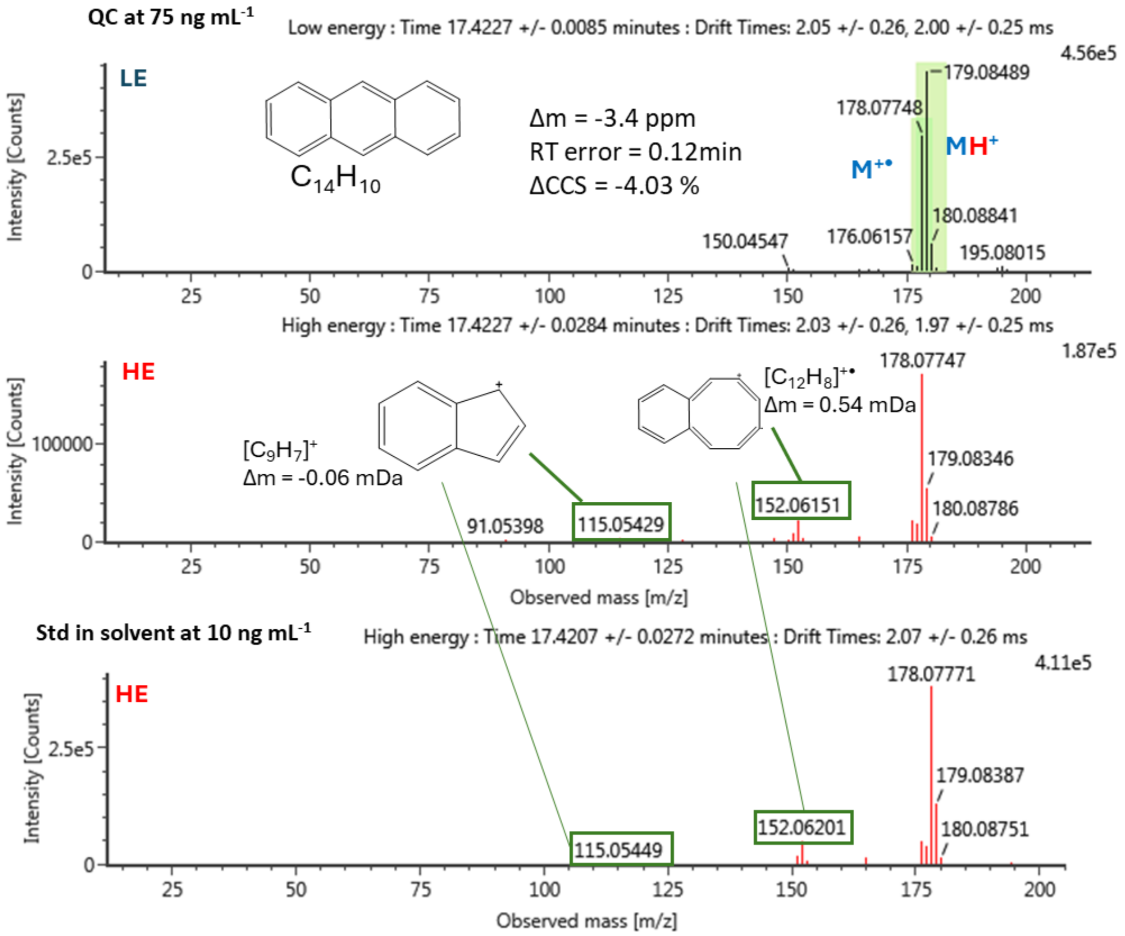

Figure S8. LE and HE spectra of anthracene in a spiked sample (ingredient 5) and HE spectra in the solvent, green squares indicate fragment ions from the analyte proposed structures for the fragment ions are displayed.

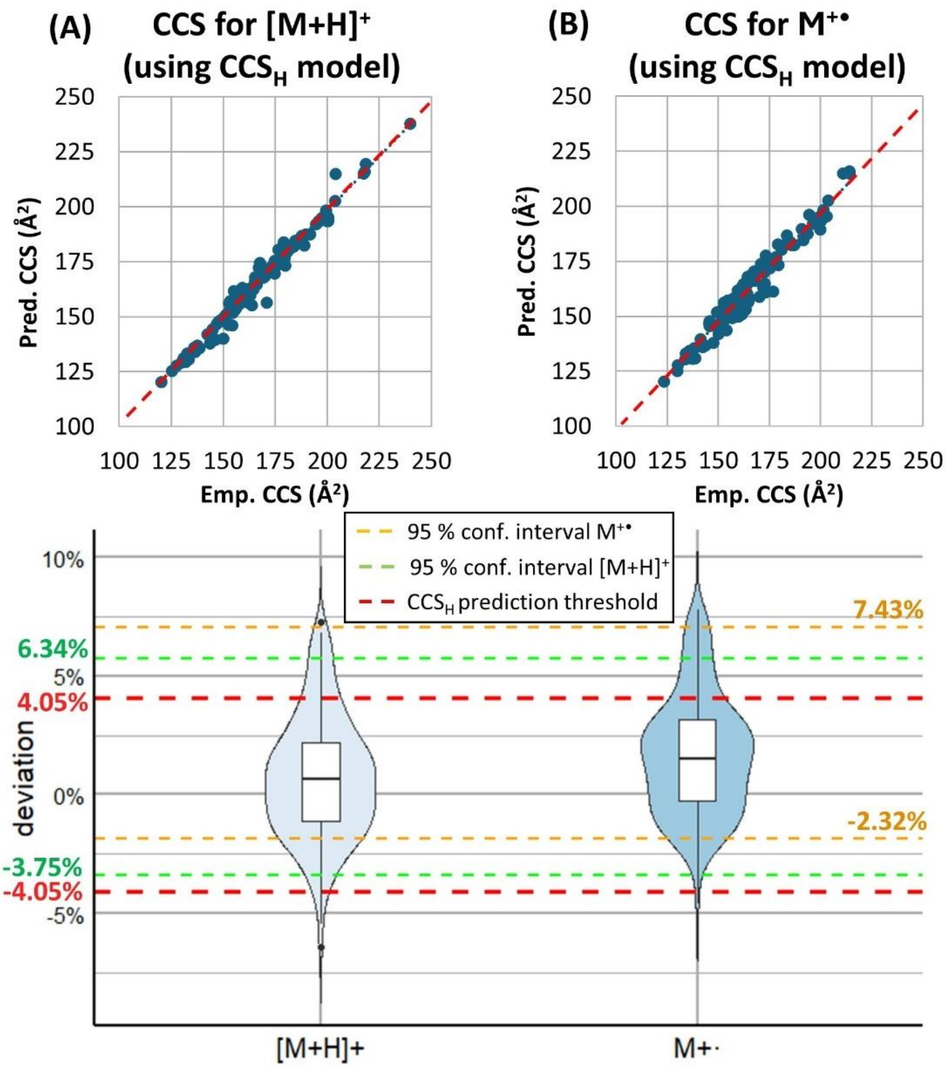

Figure S9. Top: Comparison of experimental and predicted (A) CCS for protonated using the CCS_H model and (B) CCS for molecular ions using the CCS_H model. Bottom: Violin plot of deviations between experimental and predicted data for CCS for protonated molecules using the CCS_H model. Dashed green and orange lines encompass 95% of the deviations for $[M+H]^+$ and $M^{+•}$, respectively. The dashed red line displays the threshold established for the CCS_H prediction model (95% confidence interval).

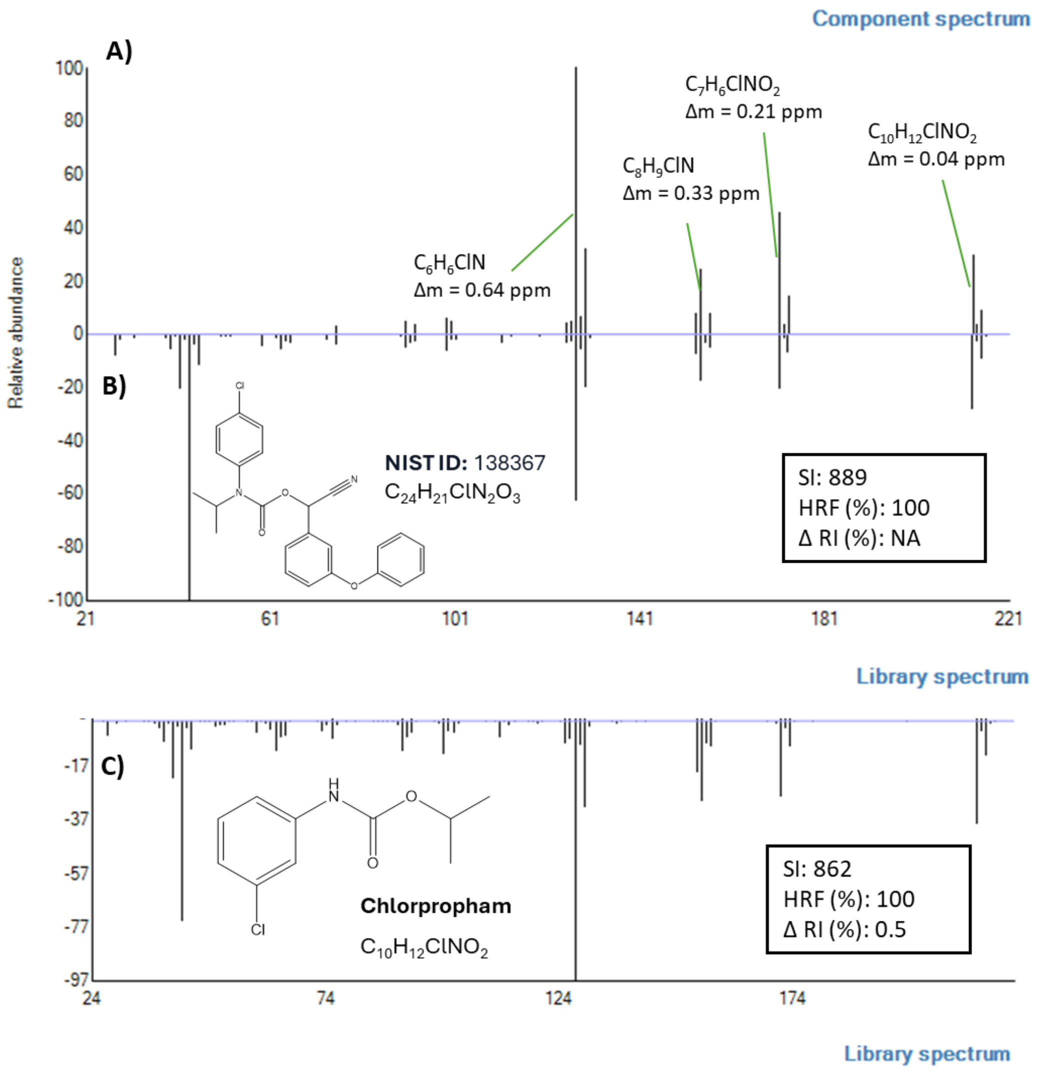

Figure S10. Experimental EI spectrum acquired with GC-Q-Orbitrap MS (A), NIST spectrum for NIST ID 138367 (B), and chlorpropham (C). Peaks used for calculating the HRF factor are marked with a green line .

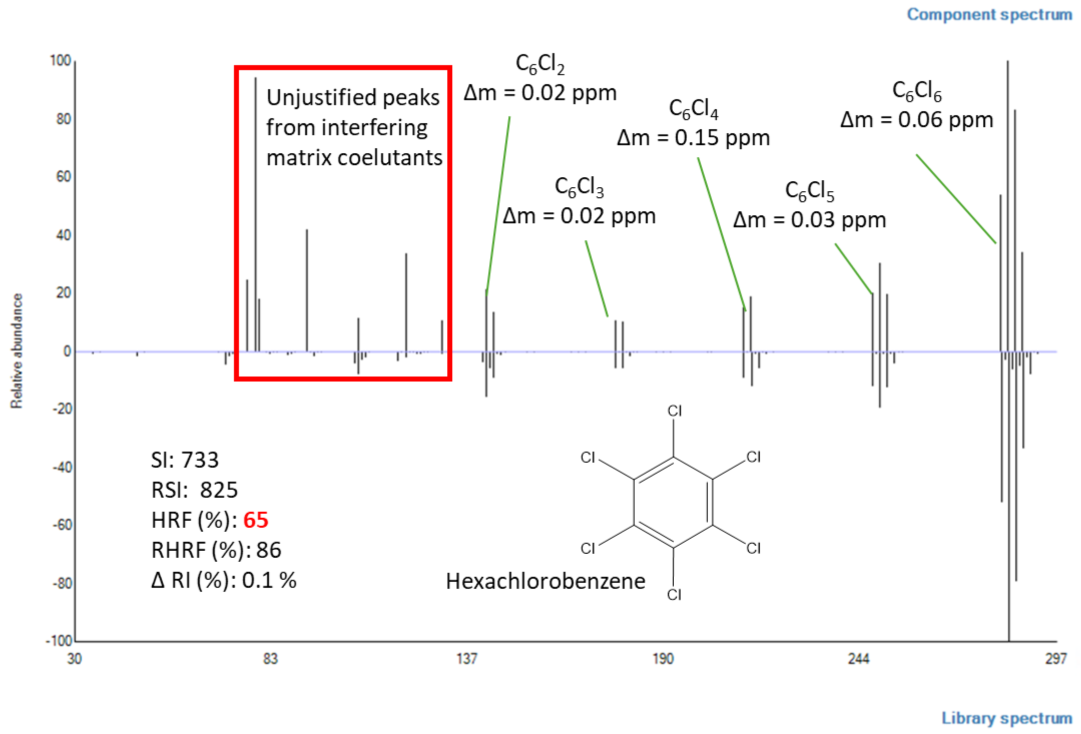

Figure S11. Comparison of experimental EI spectrum acquired with GC-Q-Orbirap MS (top) and NIST spectrum (bottom) for hexachlorobenzene. Peaks used for calculating the HRF factor are marked with a green line. The red box indicates the peaks that penalize the HRF factor.

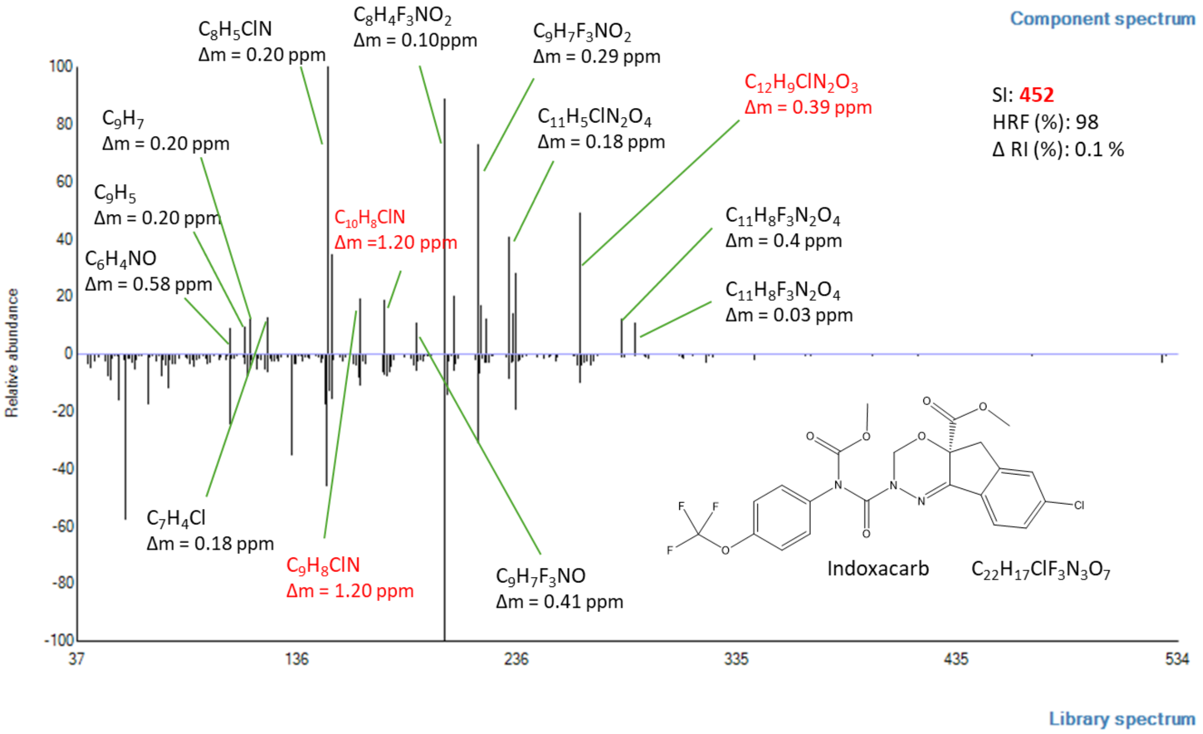

Figure S12. Comparison of experimental EI spectrum acquired with GC-Q-Orbitrap MS (top) and NIST spectrum (bottom) for indoxarb. Peaks used for calculating the HRF factor are marked with a green line. The peaks in red are fragments with Cl that do not present the correct isotopic pattern.

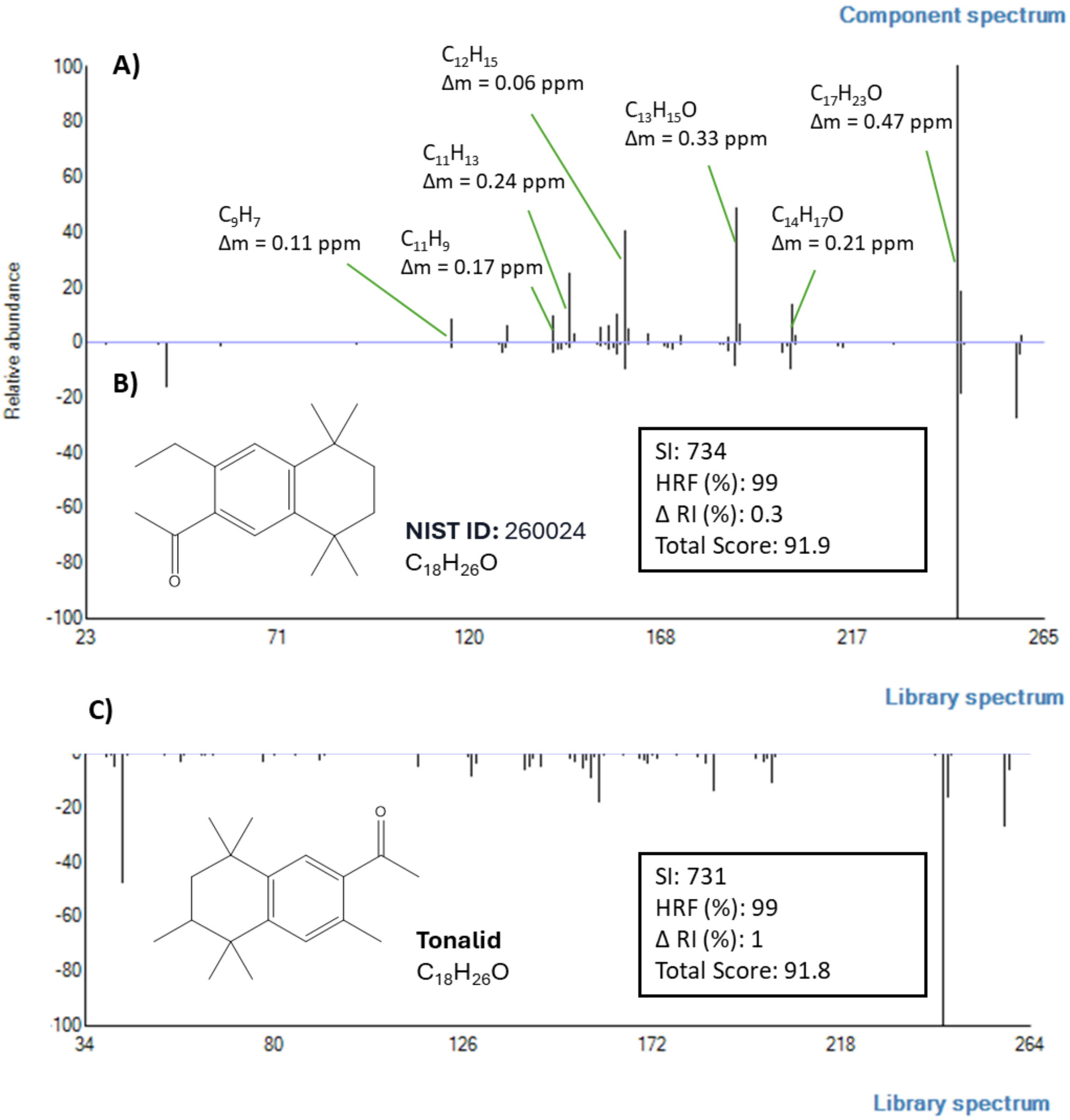

Figure S13. Experimental EI spectrum acquired with GC-Q-Orbitrap MS (A), NIST spectrum for NIST ID 260024 (B), and tonalid (C). Peaks used for calculating the HRF factor are marked with a green line.
